# The global burden of cardiovascular disease attributable to high alcohol use from 1990 to 2021: an analysis for the global burden of disease study 2021

**DOI:** 10.3389/fpubh.2025.1541641

**Published:** 2025-02-14

**Authors:** Chaofeng Niu, Juwei Dong, Peiyu Zhang, Qiwen Yang, Donghua Xue, Birong Liu, Di Xiao, Rui Zhuang, Meng Li, Lijing Zhang

**Affiliations:** Dongzhimen Hospital, Beijing University of Chinese Medicine, Beijing, China

**Keywords:** alcohol, cardiovascular diseases, mortality, disability-adjusted life years, years lived with disability, years of life lost

## Abstract

**Background:**

Cardiovascular diseases (CVDs) are the leading global disease burden, with alcohol consumption closely linked to their occurrence. This study analyzes data from the Global Burden of Disease Study 2021 (GBD 2021) to assess the distribution and trends of high alcohol use-related CVD from 1990 to 2021 across global, regional, and national levels.

**Materials and methods:**

We used the data from the GBD 2021 to conduct stratification by region, country, gender, age, SDI, and disease type in terms of the number of deaths, age-standardized mortality rate (ASMR), disability-adjusted life years (DALYs), age-standardized rate of DALYs (ASDR), years lived with disability (YLDs), age-standardized rate of YLDs, years of life lost (YLLs), and age-standardized rate of YLLs to comprehensively assess the burden of high alcohol use-related CVD from 1990 to 2021. All statistical analyses in this study were performed using R statistical software (version 4.1.2).

**Results:**

Between 1990 and 2021, global deaths, DALYs, YLDs, and YLLs attributable to high alcohol use-related CVD showed notable variation. By 2021, global deaths had doubled compared to 1990, while ASMR, ASDR, age-standardized YLD rate, and YLL rate all declined. Eastern Europe had the highest rates in 2021. Males consistently had higher ASMR, ASDR, YLD, and YLL rates compared to females, with the highest number of deaths occurring in the 70–74 age group, and the 65–69 age group showing the highest DALYs, YLDs, and YLLs. These rates increased with age. Stroke was the most common high alcohol use-related CVD, while ischemic heart disease (IHD) was the least common.

**Conclusion:**

Between 1990 and 2021, the overall burden of high alcohol use-related CVD declined globally, though some regions experienced an increase. This highlights the need for continued public health efforts, particularly targeting high-risk regions and populations, to mitigate the impact of alcohol on cardiovascular health.

## 1 Introduction

Cardiovascular disease (CVD) is one of the leading global health burdens and poses a serious threat to human health (1). Alcohol consumption, a prevalent social behavior, is strongly linked to a variety of CVDs, with its adverse effects becoming more pronounced when intake exceeds recommended limits (2). Globally, the per capita alcohol consumption has been steadily rising over the past decades (3), with excessive drinking now recognized as a significant public health concern. According to the Global Status Report on Alcohol and Health and Treatment of Substance Use Disorders released by the World Health Organization (WHO) in 2024, alcohol consumption carries a significant health and social burden. In 2019 alone, alcohol caused 2.6 million deaths globally, accounting for 4.7% of all deaths that year.

Recent studies have increasingly demonstrated that alcohol use consumption not only directly influences the incidence and mortality of CVD but also exacerbates the disease burden indirectly by promoting the development of other risk factors, including hypertension, diabetes, and obesity (2–5). The cumulative impact of these mechanisms highlights the significant role of excessive alcohol consumption in driving the global burden of CVD (6). This not only impacts individual health but also imposes a significant socioeconomic burden on public health systems (7). Therefore, studying the relationship between alcohol consumption and CVD, and analyzing the burden of high alcohol use-related CVD, holds significant public health relevance. As such, understanding the relationship between alcohol consumption and CVD, and quantifying the associated disease burden, has important public health implications. A systematic analysis of alcohol consumption and its contribution to CVD burden can provide theoretical support for the development of effective prevention and control strategies, ultimately reducing the incidence and mortality rates of CVD and alleviating the global disease burden.

This study utilizes data from the GBD Global Burden of Disease (GBD) 2021 database, conducting a comprehensive assessment of the status and trends in high alcohol use-related CVD burden from 1990 to 2021. The findings offer valuable insights for policymakers, aiding in the improvement of disease prevention and control efforts.

## 2 Materials and methods

### 2.1 Data acquisition

The GBD is the largest and most comprehensive study to date, aimed at quantifying health losses across different regions and time periods, with the goal of improving healthcare systems and eliminating disparities. We utilized publicly available data from the GBD 2021 database to evaluate the trends in deaths, disability-adjusted life years (DALYs), years lived with disability (YLDs), and years of life lost (YLLs) associated with CVD attributable to high alcohol use from 1990 to 2021. All data were sourced from the GBD 2021 database.^1^ We collected data spanning from 1990 to 2021 across 204 countries, 21 regions, and five Socio-Demographic Index (SDI) quintiles (The division of SDI regions can be referenced from Institute for Health Metrics and Evaluation).^2^ The analysis was conducted across various dimensions, including age and gender, to provide a comprehensive evaluation. In our search, we set the GBD Estimate to “Risk factor,” with Measure specified as “Deaths, DALYs, YLDs, and YLLs.” Metric was set to both “Number” and “Rate,” Risk was designated as “High alcohol use,” and Cause was specified as “Cardiovascular diseases.” The Institutional Review Board of the University of Washington waived the review of informed consent for the GBD study. This research adheres to the STROCSS criteria (8).

### 2.2 Statistical analyses

We conducted a comprehensive and integrated analysis of the four measures (deaths, DALYs, YLDs, and YLLs) across multiple dimensions, including global distribution, regional and gender differences, estimated annual percentage change (EAPC), time trends, disease-specific distribution, age distribution, and SDI analysis. In this study, age-standardized rates (ASR) were used for all comparisons, and results were expressed as means with 95% uncertainty intervals (95% UIs). For trend analyses, *p*-values <0.05 were considered statistically significant. EAPC is widely used to measure trends in ASR over time. It is calculated using a linear regression model to determine the EAPC value and the corresponding 95% confidence intervals (95% CIs) (9). All data analysis and visualization in this study were conducted using R statistical software (version 4.1.2).

## 3 Results

### 3.1 Global burden of CVD attributable to high alcohol use

**Figure 1:**
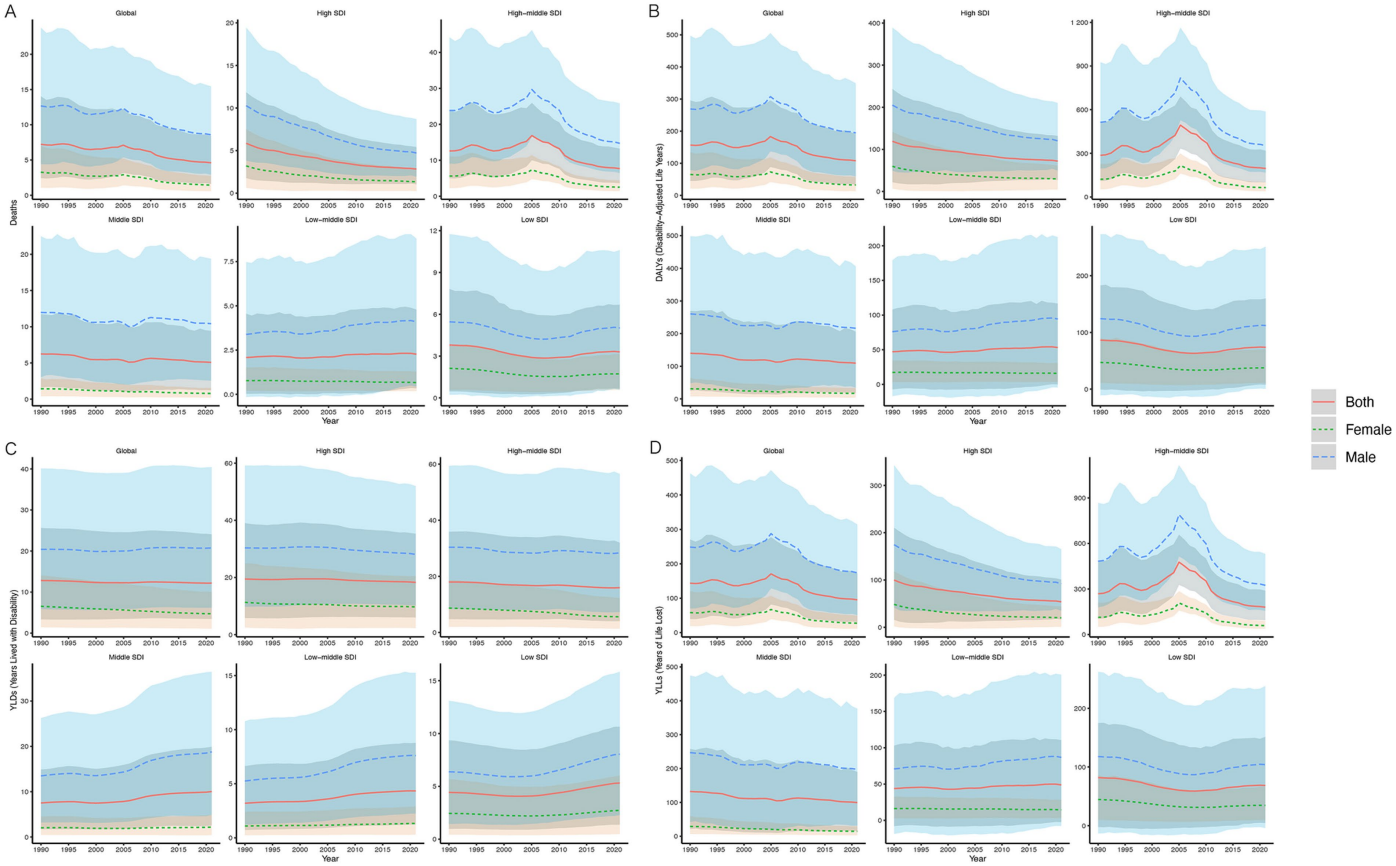
ASMR **(A)**, ASDR **(B)**, Age-Standardized Rate of YLDs **(C)**, and Age-Standardized Rate of YLLs **(D)** of high alcohol use-related CVD in both sexes combined in global and five SDI regions, 1990–2021. ASMR, age-standardized mortality rate; DALYs, disability-adjusted life years; ASDR, age-standardized rate of DALYs; YLDs, years lived with disability; YLLs, years of life lost; CVD, cardiovascular diseases; SDI, socio-demographic index.

From 1990 to 2021, the global ASMR, ASDR, age-standardized rate of YLDs, and age-standardized rate of YLLs of CVD attributable to high alcohol use have both shown an overall decreasing trend, with the decline in ASMR being the most significant, while the decrease in age-standardized rate of YLLs has been the smallest (Figure 1; Supplementary eFigure 1).

The global number of mortality decreased by 36.32% (95% UI, 21.05 to 46.81%), and the ASMR decreased from 7.23 (95% UI, 2.43 to 14.03) per 100,000 in 1990 to 4.20 (95% UI, 1.62 to 8.42) per 100,000 in 2021; the EAPC was −1.53 (95% CI, −1.77 to −1.29). Meanwhile, the number of deaths among males has consistently been higher compared to females, but the decline in mortality has been more pronounced in females, with a 55.86% (95% UI, 37.69 to 63.39%) decrease compared to a 32.30% (95% UI, 17.79 to 46.82%) decrease in males (Figure 1).

Over the past 32 years, the ASDR decreased from 156.47 (95% UI, 44.83 to 295.60) per 100,000 in 1990 to 107.59 (95% UI, 33.46–191.01) per 100,000 in 2021, with an EAPC of −1.31 (95% CI, −1.71 to −0.90). And the age-standardized rate of YLLs decreased from 143.64 (95% UI, 39.60 to 270.64) per 100,000 in 1990 to 95.41 (95% UI, 30.03 to 172.02) per 100,000 in 2021, with an EAPC of −1.43 (95% CI, −1.87 to 0.98; Figure 1; Supplementary eTable 1).

In contrast, the age-standardized rate of YLDs showed a slower decline, decreasing from 12.82 (95% UI, 3.33 to 25.63) per 100,000 in 1990 to 12.18 (95% UI, 3.48–24.06) per 100,000 in 2021, with an EAPC of −0.13 (95% CI, −0.17 to −0.09; Figure 1; Supplementary eTable 1).

### 3.2 Regional and national burden of CVD attributable to high alcohol use

**Figure 2:**
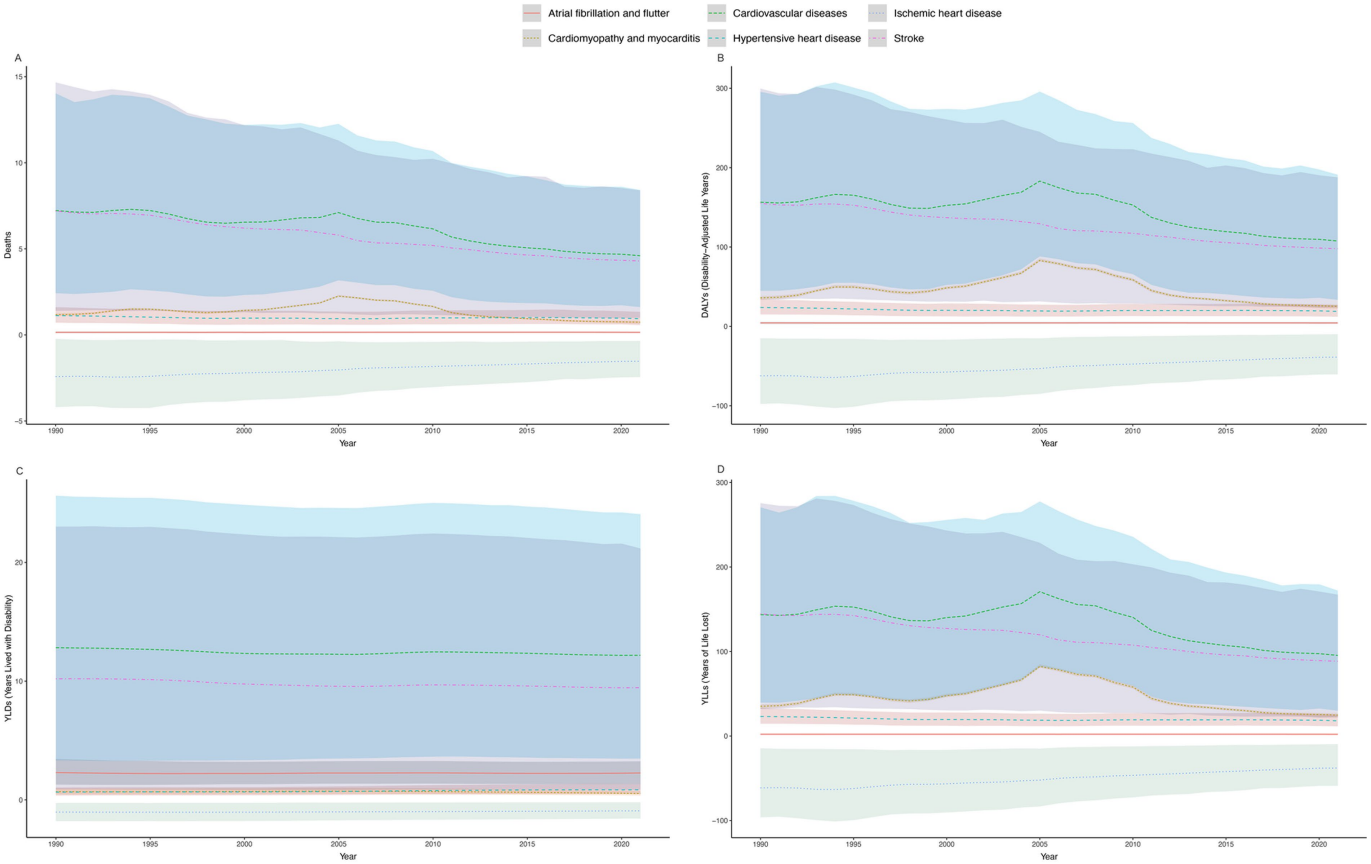
ASMR **(A)**, ASDR **(B)**, Age-Standardized Rate of YLDs **(C)**, and Age-Standardized Rate of YLLs **(D)** of high alcohol use-related CVD in both sexes combined in 204 countries and regions worldwide in 2021. ASMR, age-standardized mortality rate; DALYs, disability-adjusted life years; ASDR, age-standardized rate of DALYs; YLDs, years lived with disability; YLLs, years of life lost; CVD, cardiovascular diseases.

This study included 204 countries and regions. In 2021, the five countries with the highest ASMRs for CVD attributable to high alcohol use were the Republic of Bulgaria [26.42 (95% UI, 11.03 to 45.13) per 100,000], Republic of Latvia [23.30 (95% UI, 16.44 to 33.81) per 100,000], North Macedonia [19.22 (95% UI, 4.85 to 38.46) per 100,000], Russian Federation [18.57 (95% UI, 13.62 to 25.63) per 100,000], and Montenegro [16.75 (95% UI, 5.53 to 31.66) per 100,000]. Conversely, the Republic of Sudan [0.07 (95% UI, 0.01 to 0.16) per 100,000], American Samoa [0.05 (95% UI, −0.59 to 0.95) per 100,000], Islamic Republic of Mauritania [0.05 (95% UI, 0 to 0.12) per 100,000], Federal Republic of Somalia [0.002 (95% UI, 0 to 0.01) per 100,000], and Republic of Singapore [−0.20 (95% UI, −0.70 to 0.30) per 100,000] recorded the lowest ASMRs (Figure 2; Supplementary eTable 2).

The Republic of Latvia [747.48 (95% UI, 569.51 to 974.32) per 100,000] had the highest ASDR, followed by the Russian Federation [640.52 (95% UI, 500.00 to 815.84) per 100,000], Ukraine [544.00 (95% UI, 365.47 to 785.98) per 100,000], Republic of Bulgaria [500.74 (95% UI, 188.86 to 859.90) per 100,000], and Socialist Republic of Viet Nam [373.55 (95% UI, 80.21 to 688.37) per 100,000]. In contrast, the Republic of El Salvador [−5.60 (95% UI, −36.63 to 25.40) per 100,000] had the lowest rate (Figure 2; Supplementary eTable 2).

And the Republic of Latvia [29.09 (95% UI, 8.78 to 58.97) per 100,000] had the highest age-standardized rate of YLDs, while the Republic of Sudan [0.04 (95% UI, 0.01 to 0.06) per 100,000] had the lowest (Figure 2; Supplementary eTable 2).

At the same time, the Republic of Latvia [718.39 (95% UI, 554.23 to 926.01) per 100,000] also had the highest age-standardized rate of YLLs, while the Republic of Singapore [−7.87 (95% UI, −23.41 to 6.10) per 100,000] had the lowest (Figure 2; Supplementary eTable 2).

**Figure 3:**
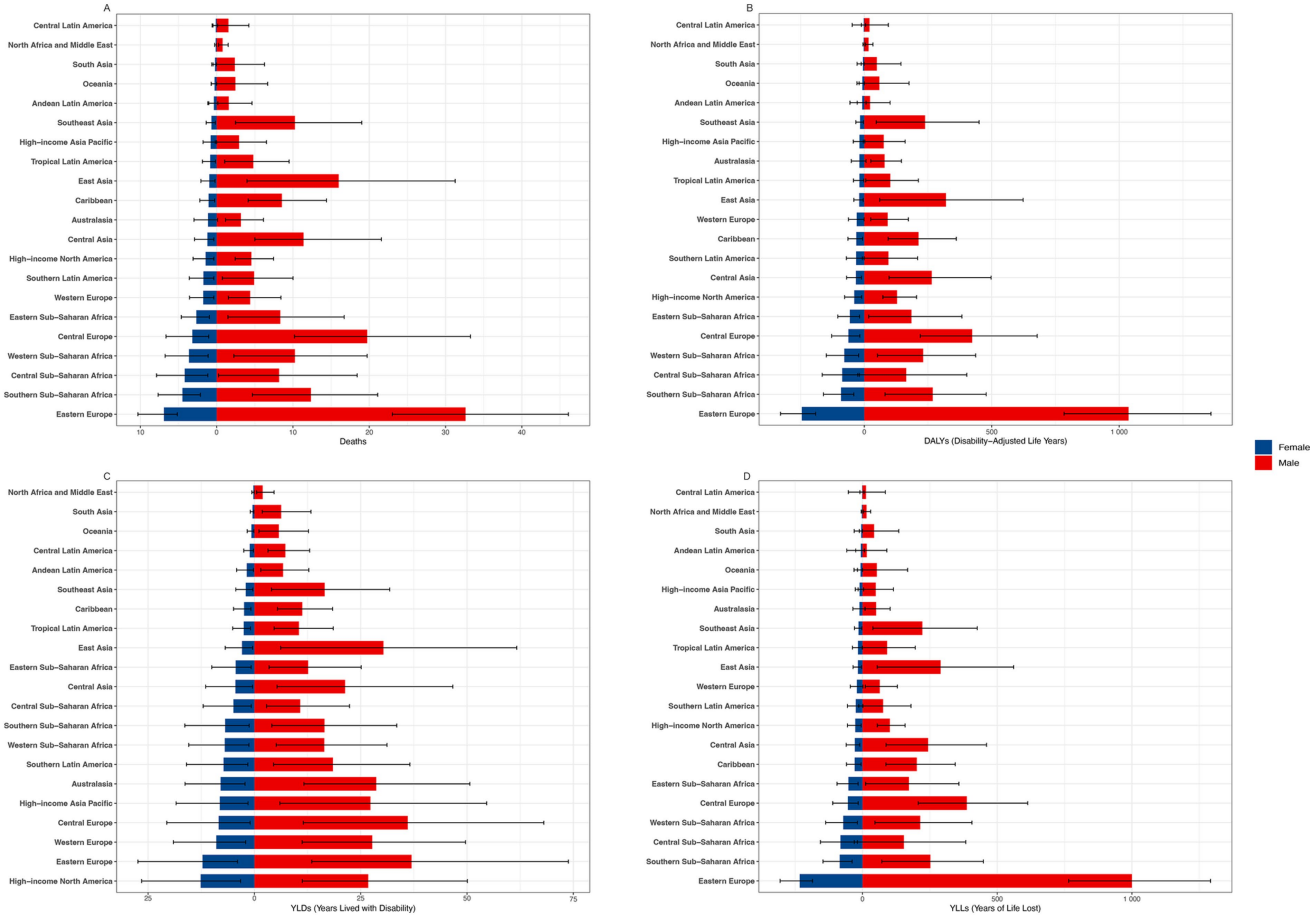
ASMR **(A)**, ASDR **(B)**, Age-Standardized Rate of YLDs **(C)**, and Age-Standardized Rate of YLLs **(D)** of high alcohol use-related CVD in males and females across 21 GBD regions in 2021. ASMR, age-standardized mortality rate; DALYs, disability-adjusted life years; ASDR, age-standardized rate of DALYs; YLDs, years lived with disability; YLLs, years of life lost; CVD, cardiovascular diseases.

In 2021, there were significant regional disparities in the ASMR, ASDR, age-standardized rate of YLDs, and age-standardized rate of YLLs of CVD attributable to high alcohol use across the 21 GBD regions (Figure 3; Supplementary eTable 1).

Eastern Europe and Central Europe had the highest ASMR, while North Africa and the Middle East had the lowest. From 1990 to 2021, the ASMR increased in 5 out of the 21 GBD regions, with Southeast Asia showing the most significant rise [EAPC of 3.50 (95% CI, 3.17–3.83)]. Conversely, mortality rates decreased in 16 regions, with the most notable decline observed in High-income Asia Pacific [EAPC of −5.11 (95% CI, −5.40 to −4.81); Figure 3; Supplementary eTable 1].

For the ASDR, 14 regions experienced a decline, with Central Latin America showing the most significant decrease [EAPC of −5.02 (95% CI, −5.41 to −4.64)]. Conversely, Southeast Asia exhibited the most pronounced increase [EAPC of 3.50 (95% CI, 3.17 to 3.83)]. Southern Sub-Saharan Africa showed the most stable trend over the past 32 years, with an EAPC of −0.01 (95% CI, −0.44 to 0.42; Supplementary eTable 1).

Eastern Europe had the highest ASDR in both 1990 and 2021, with values of 464.45 (95% UI, 277.21 to 747.24) per 100,000 and 590.80 (95% UI, 456.40 to 764.36) per 100,000, significantly higher than other regions. In contrast, North Africa and the Middle East, as well as Central Latin America, had the lowest rates in 2021, both at 9.74 (95% UI, 2.53 to 19.69) per 100,000 and 9.74 (95% UI, −25.21 to 49.23) per 100,000, respectively (Supplementary eTable 1).

Nine regions showed an increasing trend in age-standardized rate of YLDs, with Southeast Asia experiencing the most significant rise [EAPC of 2.72 (95% CI, 2.57 to 2.88)]. Conversely, 12 regions exhibited a declining trend, with Southern Latin America showing the most notable decrease [EAPC of −1.89 (95% CI, −2.04 to −1.74)]. In 2021, Eastern Europe had the highest age-standardized rate of YLDs at 22.30 (95% UI, 7.79 to 45.75) per 100,000, while North Africa and the Middle East had the lowest at 1.11 (95% UI, 0.30 to 2.65) per 100,000 (Supplementary eTable 1).

Over the past 32 years, 13 regions have shown a decline in age-standardized rate of YLLs, with Central Latin America experiencing the most significant reduction [EAPC of −6.39 (95% CI, −6.92 to −5.86)]. In contrast, eight regions have exhibited an upward trend, with Southeast Asia recording the most substantial increase [EAPC of 3.56 (95% CI, 3.22 to 3.91)]. In 2021, Eastern Europe had the highest age-standardized rate of YLLs at 22.30 (95% UI, 7.79–45.75) per 100,000, while North Africa and the Middle East recorded the lowest rate at 1.11 (95% UI, 0.30–2.65) per 100,000 (Supplementary eTable 1).

Overall, Southeast Asia demonstrated the highest increasing trends across multiple metrics, including the ASMR, ASDR, age-standardized rate of YLDs and age-standardized rate of YLLs. Conversely, High-income Asia Pacific exhibited significant declining trends across these same measures (Supplementary eTable 1).

### 3.3 Gender and age distribution trends of CVD attributable to high alcohol use

From 1990 to 2021, both globally and across the 21 GBD regions, males consistently had significantly higher ASMR, ASDR, age-standardized rate of YLDs, and age-standardized rate of YLLs for CVD attributable to high alcohol use compared to females (Figure 3; Supplementary eTable 3).

In Eastern Europe, women had the highest ASMR, ASDR and age-standardized rate of YLLs among the 21 GBD regions, with the age-standardized rate of YLDs second only to those in High-Income North America. Conversely, women in Central Latin America exhibited the lowest ASMR, ASDR and age-standardized rate of YLLs among the 21 GBD regions, with North Africa and the Middle East having the lowest age-standardized rate of YLDs for women (Figure 3; Supplementary eTable 3).

**Figure 4:**
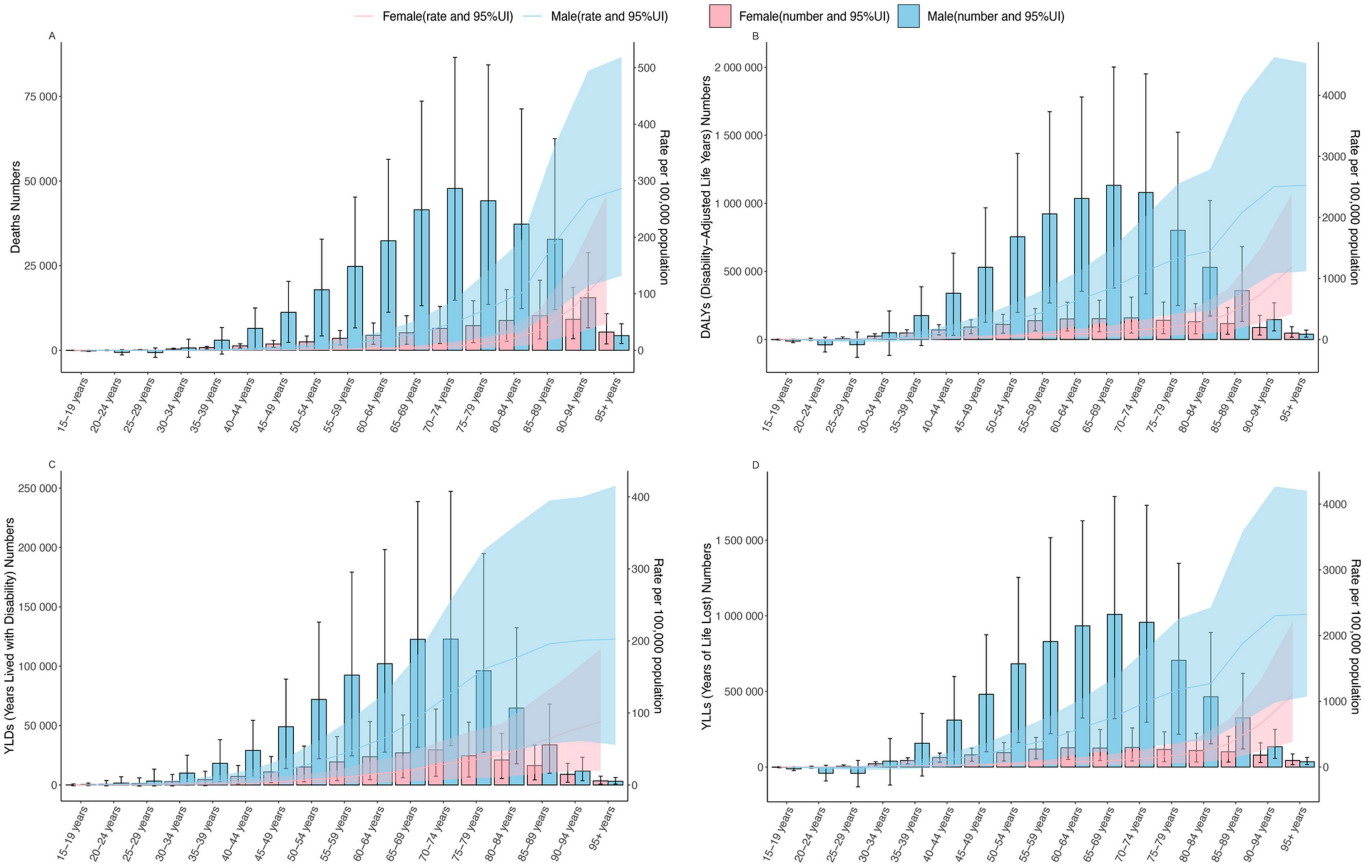
The age-specific burden of high alcohol use-related CVD, death and ASMR **(A)**, DALYs and ASDR **(B)**, YLDs and Age-Standardized Rate of YLDs **(C)**, YLLs and Age-Standardized Rate of YLLs **(D)** globally in 2021. ASMR, age-standardized mortality rate; DALYs, disability-adjusted life years; ASDR, age-standardized rate of DALYs; YLDs, years lived with disability; YLLs, years of life lost; CVD, cardiovascular diseases; UI, uncertainty interval.

In 2021, the number of deaths, DALYs, YLDs, and YLLs for CVD attributable to high alcohol use in both males and females first increased with age and then decreased. The vast majority of deaths occurred between the ages of 60 and 89, with males peaking in the 70–74 age group, while females peaked in the 85–89 age group. DALYs were most concentrated in the 40–89 age range, with males peaking in the 65–69 age group and females in the 70–74 age group. YLDs were mostly concentrated between the ages of 45 and 89, with both males and females peaking in the 70–74 age group. YLLs were also predominantly seen between the ages of 45 and 89, with males peaking in the 65–69 age group. In contrast, females showed a more distributed peak across the 55–59, 60–64, 65–69 and 70–74 age groups, with the highest concentration in the 70–74 age group (Figure 4; Supplementary eTable 4).

In 2021, the ASMR began to increase exponentially starting at age 30 for males and age 25 for females. For males, the rate of increase was most rapid between the ages of 80 and 94, followed by a deceleration in the rate of increase after age 95. Conversely, females experienced a progressively accelerating increase in ASMR with advancing age, reaching the highest rate of growth in the 95+ age group (Figure 4; Supplementary eTable 5).

For the ASDR, males exhibited an exponential increase starting at age 30, while for females, this increase began at age 25. Among males, the rate of increase was most pronounced between the ages of 80 and 94, after which it gradually plateaued. In contrast, for females, the rate of increase continued to accelerate with age, reaching its peak after age 95 (Figure 4; Supplementary eTable 5).

The age-standardized rate of YLDs exhibited an exponential increase beginning at age 15 for males and age 20 for females. In males, the rate of increase was most rapid between the ages of 60 and 79, whereas in females, the most significant acceleration occurred after age 80 (Figure 4; Supplementary eTable 5).

For age-standardized rate of YLLs, an exponential increase in ASMR was observed starting at age 30 for males and age 25 for females. Among males, the rate of increase was most rapid between the ages of 80 and 89, while for females, the fastest growth occurred after age 90 (Figure 4; Supplementary eTable 5).

### 3.4 Trends in the distribution of disease types of CVD attributable to high alcohol use

**Figure 5:**
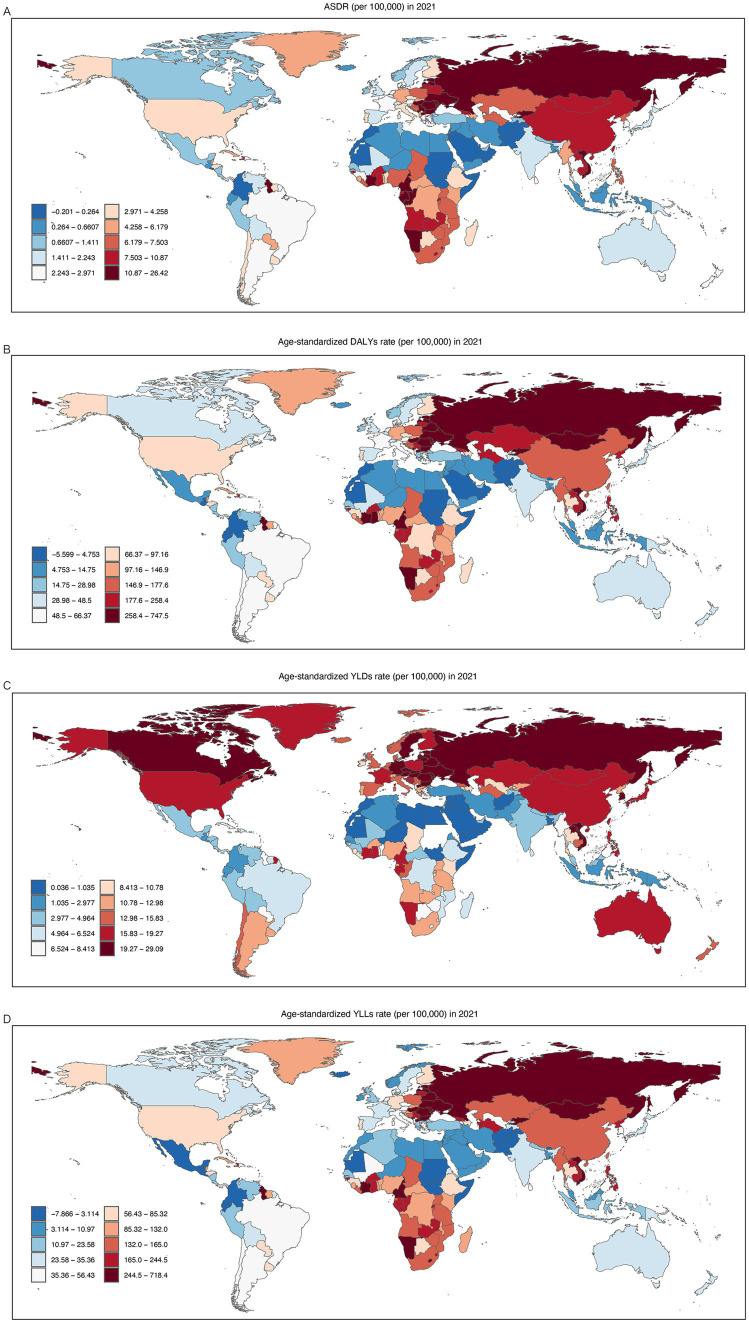
ASMR **(A)**, ASDR **(B)**, Age-Standardized Rate of YLDs **(C)**, and Age-Standardized Rate of YLLs **(D)** for five types of high alcohol use-related CVD in both sexes combined globally, 1990–2021. ASMR, age-standardized mortality rate; DALYs, disability-adjusted life years; ASDR, age-standardized rate of DALYs; YLDs, years lived with disability; YLLs, years of life lost; CVD, cardiovascular diseases.

According to the GBD database, CVD include five categories: Atrial fibrillation and flutter, Cardiomyopathy and myocarditis, Hypertensive heart disease (HHD), ischemic heart disease (IHD) and Stroke. From 1990 to 2021, Stroke consistently held the highest position in terms of ASMR, ASDR, age-standardized rate of YLDs and age-standardized rate of YLLs. Conversely, IHD consistently recorded the lowest rates across these metrics. Notably, Atrial fibrillation and flutter showed and HHD minimal change over the 30-year period (Figure 5; Supplementary eFigure 2).

It is noteworthy that the ASMR for cardiomyopathy and myocarditis has been decreasing annually since 2005. By 2014, it had fallen below that of HHD, making it the third leading cause of death among CVD (Figure 5; Supplementary eTable 6).

Among the 21 GBD regions, Central Europe, Eastern Europe, East Asia, Western Sub-Saharan Africa, Central Sub-Saharan Africa and Southern Sub-Saharan Africa rank among the top in stroke of ASMR, ASDR, age-standardized rate of YLDs and age-standardized rate of YLLs (Figure 4).

### 3.5 Trends in the SDI distribution of CVD attributable to high alcohol use

In 2021, the number of CVD attributable to high alcohol use deaths and ASMR in the High-Middle SDI region were the highest, at 147,629.02 (95% UI, 68,211.77 to 256,194.13) and 7.68 (95% UI, 3.60 to 13.24) per 100,000, respectively. Between 1990 and 2021, the High SDI region experienced the largest decline in death cases, with a reduction of −51.63% (95% UI, −68.12 to −26.68) and an EAPC of −2.32% (95% CI, −2.43 to −2.21). Among the five SDI regions, only the Low-Middle SDI region saw an increase in death cases, with an EAPC of 0.37 (95% CI, 0.28 to 0.46; Supplementary eTable 1).

For ASDR, between 1990 and 2021, the Middle SDI region experienced the largest decline, with an EAPC of −0.67 (95% CI, −0.81 to −0.53). Conversely, only the Low-Middle SDI region saw an increase, with an EAPC of 0.48% (95% CI: 0.37 to 0.58; Supplementary eFigure 3; Supplementary eTable 1).

Between 1990 and 2021, only the High SDI and High-Middle SDI regions saw a reduction in age-standardized rate of YLDs. The High SDI region experienced the largest decrease in age-standardized rate of YLLs, with an EAPC of −1.91 (95% CI, −2.00 to −1.82; Supplementary eFigure 3; Supplementary eTable 1).

## 4 Discussion

CVD remains a leading cause of death and disability globally (10), and high alcohol use-related premature mortality and disability have far-reaching effects on individuals, families, and society. Currently, over 2 billion individuals aged 15 and older worldwide consume alcohol, representing 43% of the global population (11). This means that roughly one in every three people is a current drinker. In recent years, as research into the link between alcohol consumption and CVD has deepened, increasing evidence shows a strong association between alcohol intake and the development of CVD. The consensus is growing that the less alcohol consumed, the better for cardiovascular health (12–15). However, due to differences in population distribution, economic levels, and significant variations in alcohol consumption policies among countries, the health burden caused by alcohol consumption varies significantly between countries and regions. In this study, we systematically evaluated the changes in the global burden of CVD attributable to high alcohol use from 1990 to 2021. By analyzing data from the 2021 GBD database, we found significant variations in the distribution of high alcohol use-related CVD across different genders, age groups, time periods, regions, and types of diseases. These disparities highlight the complex impact of high alcohol consumption on cardiovascular health across diverse populations.

### 4.1 Analysis of the global burden of CVD attributable to high alcohol use

In this study, we systematically evaluated the changes in the global burden of CVD attributable to high alcohol use from 1990 to 2021. By analyzing data from the 2021 GBD database, we found significant variations in the distribution of high alcohol use-related CVD across different genders, age groups, time periods, regions, and types of diseases. These disparities highlight the complex impact of high alcohol consumption on cardiovascular health across diverse populations.

Globally, the number of CVD deaths due to high alcohol use increased from 253,061.89 in 1990 to 385,825.09 in 2021, reflecting a 52.46% rise. This may be linked to substantial global population growth, accelerated population aging (16). However, the ASMR decreased from 7.23 to 4.60. At the same time, the global ASMR, ASDR, age-standardized rate of YLDs, and age-standardized rate of YLLs all showed a decline over the 32-year period. These results are consistent with the Global Status Report on Alcohol and Health and Treatment of Substance Use Disorders released by WHO in 2024. This decline may be associated with the global strategy adopted by the WHO to reduce alcohol consumption. In 2010, the WHO called for a relative reduction of at least 10% in the harmful use of alcohol by 2025 at the national level, with a further relative reduction of at least 20% by 2030 compared to 2010 levels. As a result, global per capita alcohol consumption decreased slightly from 5.7 liters in 2010 to 5.5 liters in 2019, representing a relative reduction of 4.5%.

In terms of gender distribution, males exhibit significantly higher ASMR, ASDR, age-standardized rate of YLDs, and age-standardized rate of YLLs compared to females. However Furthermore, the EAPC for females has decreased more than that for males. In 2021, the ASMR for men (8.58 per 100,000) was approximately six times higher than that for women (1.44 per 100,000), and the EAPC for women was about twice that of men. This is primarily due to the fact that alcohol consumption in men is substantially higher, while many women drink less or abstain from alcohol entirely throughout their lives (17, 18). According to WHO data from 2024, 45% of men and 27% of women worldwide engage in heavy episodic drinking (HED), defined as consuming at least 60 grams of pure alcohol in a single occasion within the past month. Additionally, while men generally consume much larger quantities of alcohol, many women drink less or abstain entirely. In 2019, 3.6% of adults were classified as engaging in heavy continuous drinking (HCD), defined as consuming more than 60 grams of alcohol per day on average. Among men, the prevalence of HCD was 6.7%, whereas the prevalence in women was much lower, at 0.6%. Additionally, this pattern may also be related to the drinking habits of men, including long-term, repeated binge drinking and a preference for strong spirits (5). Men are more likely to engage in binge drinking and chronic alcohol use, which significantly increases their risk of developing CVD. These drinking behaviors can exacerbate the negative effects of alcohol on the heart and blood vessels, further contributing to the higher burden of high alcohol use-related CVD in men compared to women (15, 19).

Alcohol consumption is often regarded as a marker of adulthood, with the vast majority of drinkers being young adults. In 2019, individuals aged 15 to 19 accounted for 22.0% of the total drinking population (11). The study results indicate that high alcohol use-related CVD predominantly occur in individuals over the age of 50, with the highest number of deaths observed in the 70–74 age group. Current research suggests that this trend may be attributed to several factors: a higher prevalence of underlying health conditions in older adults, higher alcohol consumption in this demographic (20, 21), and the greater physical damage alcohol causes compared to younger populations (22). As people age, the likelihood of having chronic conditions such as hypertension, diabetes, and hyperlipidemia significantly increases (23). These conditions are major risk factors for CVD on their own, and when combined with long-term alcohol consumption, they further strain the cardiovascular system, increasing the risk of adverse events (24). Additionally, middle-aged and older adults may maintain higher levels of alcohol consumption due to cumulative lifestyle habits and social influences (25, 26). Compared to younger people, their ability to metabolize alcohol decreases, and their liver, kidneys, and cardiovascular systems become more sensitive to alcohol’s harmful effects (27).

### 4.2 Analysis of the regional and national burden of CVD attributable to high alcohol use

Over the past 32 years, only High SDI regions have shown a consistent annual decline in the ASMR, ASDR, age-standardized rate of YLDs, and age-standardized rate of YLLs for high alcohol use-related CVD. In contrast, High-middle, Middle, and Low SDI regions exhibited fluctuating trends, with the greatest variability observed in High-middle SDI regions. Interestingly, although Low-middle SDI regions had the lowest ASMR, ASDR, age-standardized rate of YLDs, and age-standardized rate of YLLs among the five SDI regions, they demonstrated an upward trend over time.

Generally, there is a positive correlation between SDI levels and alcohol consumption rates (28). However, we found that the ASMR for alcohol-related CVD was the lowest in low-middle SDI regions in (20212.27 [0.33 to 4.79] per 100,000). Nevertheless, this rate has shown an increasing trend over time from 1990 to 2021. This discrepancy may primarily be associated with differences in economic development, public health policies, and lifestyle factors across regions. In 2010, the WHO released a global strategy to reduce the harmful use of alcohol, providing governments with intervention measures (29, 30). By 2019, 56% of countries had established formal national alcohol policies, with the greatest contribution coming from middle-income countries. However, the recognition and implementation of this strategy have varied significantly across different countries and regions (31). In High-SDI regions, growing awareness of alcohol’s harmful effects has led to the implementation of strict policies, such as increased alcohol taxes, advertising restrictions, and tighter control over underage drinking, which have effectively reduced alcohol misuse (32). Moreover, the widespread dissemination of health education has heightened public awareness of the risks associated with alcohol consumption, prompting many individuals to reduce or abstain from drinking altogether (33). Most importantly, High-SDI regions benefit from well-established healthcare systems and sufficient medical resources, allowing for better prevention, diagnosis, and treatment of high alcohol use-related CVD.

In Low-middle SDI regions, significant economic growth and accelerated urbanization in recent years have led to substantial lifestyle changes, including an increase in social events, alcohol consumption, and the intake of high-salt and high-fat diets (34). This has resulted in a rapid rise in the incidence of CVD. Additionally, these regions often face challenges such as limited healthcare resources, more lenient alcohol consumption controls, and an aging population, all of which contribute to the increasing burden of high alcohol use-related CVD. Therefore, policymakers and governmental agencies in various countries must prioritize and reassess alcohol control policies and health programs to effectively reduce the health burden associated with alcohol-related cardiovascular diseases.

In 2021, across the 21 regions, Eastern Europe exhibited the highest overall ASMR, ASDR, age-standardized rate of YLDs, and age-standardized rate of YLLs. Among women, High-Income North America showed the highest age-standardized rate of YLDs. North Africa and the Middle East had the lowest ASMR, ASDR, and age-standardized rate of YLDs, while Central Latin America had the lowest age-standardized rate of YLLs.

These findings are closely related to dietary habits, lifestyle choices, and public health conditions in these regions. The WHO has pointed out that in recent years, Europe has made very little progress in reducing alcohol consumption and its related harms. Eastern Europe is among the regions with the highest alcohol consumption globally, particularly for hard liquors. Despite various efforts and strategies aimed at curbing harmful drinking behaviors, alcohol use remains a significant public health issue in the region. The slow progress may be attributed to cultural factors, the accessibility and social acceptance of alcohol, as well as challenges in implementing and enforcing effective alcohol control measures across European countries. This significantly increases the incidence of high alcohol use-related CVD, leading to elevated ASMR, DALYs, and YLLs. Additionally, diets in many Eastern European countries are typically high in fat and salt, and smoking rates remain high, further driving cardiovascular disease prevalence (35, 36). Furthermore, economic instability and social stress in some countries of the region may have contributed to rising mental health issues. Long-term socioeconomic pressure is a well-known risk factor for chronic diseases such as hypertension and heart disease, compounding the high mortality and disability rates (37).

In High-Income North America, advanced medical care extends the lifespan of patients with chronic diseases, but postmenopausal women, lacking the protective effects of estrogen, face increased cardiovascular risk (38). Women in this region, particularly those balancing work and family responsibilities, may also experience heightened stress, contributing to the highest global age-standardized rate of YLDs among women.

In North Africa and the Middle East, a relatively young population structure and lower levels of aging contribute to reduced rates of chronic diseases and associated mortality. Additionally, many countries in this region have a majority Muslim population, and Islamic law prohibits the production, consumption, transportation, and trade of alcohol. As a result, the per capita alcohol consumption in this region is among the lowest globally, plays a key role in keeping these health indicators low (39, 40). Despite recent shifts toward modernized lifestyles, the traditional Mediterranean diet, rich in vegetables, fish, and olive oil, prevalent in parts of the Middle East and North Africa, has helped mitigate the risk of CVD. At the same time, countries in the North Africa and the Middle East region have been actively implementing reforms to upgrade their healthcare systems, and significant progress has been made in recent years (41).

In Central Latin America, healthcare systems have improved over the past few decades, particularly in the early diagnosis and treatment of chronic diseases, resulting in fewer premature deaths and lower YLLs. In countries like Chile and Costa Rica, enhancements in public health policies, such as prevention measures for hypertension, diabetes, and obesity, have made early management of CVD more effective, thus reducing the rate of life years lost.

According to our research findings, the risk of high alcohol use-related CVD varies across different regions and is closely associated with factors such as regional economies, healthcare systems, dietary habits, and religious beliefs. As awareness of the harmful effects of alcohol on health continues to grow, the overall burden of high alcohol use-related CVD has decreased, although significant disparities remain between countries and regions. The WHO should continue to urge all nations to intensify their efforts, tailoring policies to specific national conditions and cultural contexts. Implementing alcohol control measures based on local circumstances, combined with education, policy interventions, and clinical support, will be key strategies in reducing the burden of high alcohol use-related CVD.

### 4.3 Analysis of disease type distribution of CVD attributable to high alcohol use

In the 2021 GBD database, the five major categories of CVD exhibited varying trends across different metrics. Stroke consistently ranked highest in terms of ASMR, ASDR, age-standardized rate of YLDs, and age-standardized rate of YLLs over the past 32 years, although its rates have been gradually declining. In terms of ASMR, ASDR, and age-standardized rate of YLLs, the trends for all five diseases were generally consistent, with Cardiomyopathy and myocarditis showing the greatest variability. HHD exhibited a slow upward trend and surpassed Cardiomyopathy and myocarditis in 2013 to become the second leading cause of high alcohol use-related CVD. Interestingly, IHD has remained consistently the lowest in terms of high alcohol use-related CVD throughout this period. Regarding age-standardized rate of YLDs, the trends for all five diseases have remained largely stable over the past 32 years.

Stroke is a leading cause of death and severe disability globally, particularly among the middle-aged and older adults. In 2021, approximately 7.3 million (6.6 to 7.8 million) deaths were attributed to stroke, accounting for 10.7% (9.8–11.3%) of all deaths globally (42). Alcohol consumption increases the risk of stroke, with heavy drinking contributing to 9.5% of stroke-related mortality (43). The potential mechanisms include alcohol’s activation of the sympathetic nervous system and the renin-angiotensin-aldosterone system, leading to vasoconstriction and coagulation abnormalities, which increase the likelihood of stroke occurrence (44, 45). However, with improvements in global healthcare conditions, early interventions and preventive measures for stroke have been gradually strengthened, especially in developed countries and some middle-income countries (46, 47). The widespread adoption of treatments such as thrombolytic therapy and interventional surgeries has contributed to a decline in stroke-related mortality and morbidity (48). These medical advances have resulted in reductions in stroke-related ASMR, DALYs, and YLLs.

In 2021, there were 402,038 deaths globally due to cardiomyopathy and myocarditis, of which approximately 63,990 (16%) were attributed to alcohol consumption (49). The etiology of cardiomyopathy and myocarditis is diverse, encompassing infectious, immunological, and genetic causes, with numerous sudden and uncontrollable factors (50, 51). The prevalence of myocarditis can surge during epidemics or outbreaks of specific diseases, impacting mortality rates and disease burden trends. This variability explains the marked fluctuations in ASMR, ASDR, and age-standardized rate of YLLs for these conditions. Although the causes of cardiomyopathy are complex, advancements in diagnostic techniques, such as cardiac imaging and genetic testing, as well as improvements in therapeutic approaches, have extended patient survival (51). However, mortality rates still exhibit significant variability due to the unpredictable nature of some of these conditions.

With the global population aging, rising obesity rates, and the increasing prevalence of unhealthy lifestyles, the incidence of hypertension has been steadily growing year by year (52). Alcohol consumption is a significant risk factor for hypertension, with a positive correlation between drinking and the risk of developing high blood pressure (44). Studies have shown that reducing alcohol consumption leads to a decrease in blood pressure, with a dose–response relationship observed between the two (53). Excessive alcohol intake (defined as three or more drinks per day) significantly raises blood pressure, both in individuals with hypertension and in those with normal blood pressure (54). The mechanisms underlying this effect are thought to include alcohol-induced activation of the sympathetic nervous system, which causes vasoconstriction and increases cardiac contractility. Additionally, alcohol may reduce the sensitivity of arterial baroreceptors, thereby diminishing the central nervous system signals required to normalize blood pressure. Furthermore, alcohol consumption may interfere with the pharmacological effects of antihypertensive medications, potentially counteracting their blood pressure-lowering effects (55–57). This directly contributes to the upward trend in HHD. The issue is particularly pronounced in low-and middle-income countries, where hypertension management is often inadequate. Many patients remain undiagnosed and untreated until hypertension leads to complications (58). This delayed diagnosis and treatment further drive the annual increase in HHD rates.

Alcohol consumption is positively correlated with the mortality rate of IHD. After controlling for other confounding factors, drinking increases the risk of developing IHD by 1.5 to 2.0 times (59). However, among high alcohol use-related CVD, IHD has the lowest contribution. This could be because alcohol is not a primary direct risk factor for IHD, and the diagnosis and treatment of coronary heart disease have become relatively advanced and well-established (60). These factors may explain the lower proportion of IHD in high alcohol use-related CVD. With improvements in the treatment and management of CVD, the acute mortality rate has decreased. However, the number of patients requiring long-term management for chronic conditions, such as stroke sequelae and heart failure, has increased. This trend leads to a relatively stable rate of YLDs, as patients survive the acute phase but continue to live with the long-term complications of CVD.

This study has several limitations. First, much of the high alcohol use-related data in the GBD relies on self-reported drinking habits. Self-reporting of alcohol consumption is often inaccurate, with many individuals underestimating or overestimating their actual intake, leading to data bias. Second, while the study generally discusses the overall impact of “drinking,” in reality, different types of alcoholic beverages (such as beer, spirits, and wine) may have varying effects on health. The GBD database does not differentiate the specific health impacts of different beverages, resulting in generalized data processing. Third, the majority of current research, including authoritative studies, indicates that even moderate alcohol consumption is harmful to the body (61). The GBD database overall views alcohol as a negative factor, making it difficult to fully distinguish the differing health effects between moderate and excessive drinking. This could obscure the clear separation of alcohol’s positive and negative effects, particularly in cardiovascular disease research. Fourth, the data in the GBD often reflect long-term health burdens, but for certain populations, especially those with intermittent drinking behaviors, the long-term effects of alcohol may be underestimated or overestimated. For instance, occasional binge drinking and daily moderate drinking have markedly different health impacts, but the GBD database struggles to accurately distinguish these subtle differences.

In conclusion, although the overall burden of high alcohol use-related CVD is gradually declining, significant regional and gender differences persist. The annual number of cardiovascular disease deaths attributable to alcohol consumption continues to rise, particularly in high-risk regions and specific populations. Therefore, public health authorities should implement stricter alcohol control measures while enhancing health education and awareness campaigns to further reduce the burden of these diseases.

## Data Availability

The datasets presented in this study can be found in online repositories. The names of the repository/repositories and accession number(s) can be found in the article/Supplementary material.

## Footnotes

^1^ https://vizhub.healthdata.org/gbd-results/

^2^ https://ghdx.healthdata.org/search/site/SDI
