# Supplementary material for: The global burden of cardiovascular disease attributable to high alcohol use from 1990 to 2021: an analysis for the global burden of disease study 2021
Source: Front Public Health. 2025 Feb 14;13:1541641. doi: 10.3389/fpubh.2025.1541641 (PMC11868065; doi:10.3389/fpubh.2025.1541641)
Supplement: Supplementary file 1 [file Table_1.docx]

**Supplementary Documents 1**

**eFigure 1**: The EAPC of ASMR (A), ASDR (B), Age-Standardized Rate of YLDs (C), and Age-Standardized Rate of YLLs (D) of high alcohol use-related CVD in both sexes combined across 21 GBD regions, 1990-2021. EAPC, estimated annual percentage change; ASMR, age-standardized mortality rate; DALYs, disability-adjusted life years; ASDR, age-standardized rate of DALYs; YLDs, years lived with disability; YLLs, years of life lost; CVD, cardiovascular diseases.

**eFigure 2**: The heatmap of five types high alcohol use-related CVD showing ASMR (A), ASDR (B), Age-Standardized Rate of YLDs (C), and Age-Standardized Rate of YLLs (D) across 21 GBD regions in 2021. ASMR, age-standardized mortality rate; ASDR, age-standardized rate of DALYs; YLDs, years lived with disability; YLLs, years of life lost; CVD, cardiovascular diseases.

**eFigure 3**: ASMR (A), ASDR (B), Age-Standardized Rate of YLDs (C), and Age-Standardized Rate of YLLs (D) for high alcohol use-related CVD in both sexes combined across SDI regions in 2021. ASMR, age-standardized mortality rate; ASDR, age-standardized rate of DALYs; YLDs, years lived with disability; YLLs, years of life lost; CVD, cardiovascular diseases.
