# Supplementary material for: The global burden of cardiovascular disease attributable to high alcohol use from 1990 to 2021: an analysis for the global burden of disease study 2021
Source: Front Public Health. 2025 Feb 14;13:1541641. doi: 10.3389/fpubh.2025.1541641 (PMC11868065; doi:10.3389/fpubh.2025.1541641)
Supplement: Supplementary file 2 [file Table_2.docx]

**Supplementary Documents 2**

**eTable 1:** ASDR, Age-Standardized Rate of YLDs, Age-Standardized Rate of YLLs of high alcohol use-related CVD between 1990 and 2021 at the global and regional level. ASDR, age-standardized rate of DALYs; YLDs, years lived with disability; YLLs, years of life lost; CVD, cardiovascular diseases; SDI, socio-demographic index; UI, uncertainty intervals; CI, confidence interval; EAPC, estimated annual percentage change.

|  | **1990** | | | **2021** | | | **EAPC 1990-2021** | | |
| --- | --- | --- | --- | --- | --- | --- | --- | --- | --- |
|  | **ASDR**  **per 100,000, N (95% UI)** | **Age-Standardized Rate of YLDs**  **per 100,000, N (95% UI)** | **Age-Standardized Rate of YLLs**  **per 100,000, N (95% UI)** | **ASDR**  **per 100,000, N (95% UI)** | **Age-Standardized Rate of YLDs**  **per 100,000, N (95% UI)** | **Age-Standardized Rate of YLLs**  **per 100,000, N (95% UI)** | **ASDR**  **per 100,000, N (95% UI)** | **Age-Standardized Rate of YLDs**  **per 100,000, N (95% UI)** | **Age-Standardized Rate of YLLs**  **per 100,000, N (95% UI)** |
| **Global** | 156.47(44.83-295.60) | 12.82(3.33-25.63) | 143.64(39.60-270.64) | 107.59(33.46-191.01) | 12.18(3.48-24.06) | 95.41(30.03-172.02) | -1.31(-1.71--0.90) | -0.13(-0.17--0.09) | -1.43(-1.87-0.98) |
| Male | 268.94(78.80-496.66) | 20.41(5.86-40.12) | 248.53(70.31-462.04) | 194.53(59.09-349.07) | 20.75(6.21-40.44) | 173.78(51.12-314.22) | -1.12(-1.47--0.77) | 0.11(0.06-0.16) | -1.24(-1.62-0.86) |
| Female | 64.92(21.03-134.18) | 6.54(1.45-14.04) | 58.38(19.00-120.32) | 32.16(13.04-57.06) | 4.71(1.08-10.04) | 27.45(11.23-49.37) | -2.43(-2.98--1.88) | -1.10(-1.12--1.08) | -2.61(-3.21-1.99) |
| **SDI** |  |  |  |  |  |  |  |  |  |
| High SDI | 119.24(21.93-243.36) | 19.45(5.79-39.01) | 99.79(15.53-210.27) | 72.11(26.99-131.94) | 18.27(5.99-35.31) | 53.84(16.26-100.82) | -1.55(-1.62--1.48) | -0.19(-0.24-0.15) | -1.91(-2.00-1.82) |
| High-middle SDI | 286.47(110.05-515.56) | 17.99(4.76-35.90) | 268.49(103.08-483.37) | 196.03(100.33-317.67) | 15.98(4.06-32.01) | 180.05(93.90-288.56) | -1.50(-2.37-0.62) | -0.40(-0.44--0.37) | -1.58(-2.50-0.65) |
| Middle SDI | 139.87(24.54-269.79) | 7.51(1.71-14.90) | 132.36(21.18-257.81) | 109.42(17.31-205.76) | 10.04(2.56-19.92) | 99.38(15.51-189.95) | -0.67(-0.81-0.53) | 1.10(0.93-1.27) | -0.80(-0.94--0.66) |
| Low-middle SDI | 47.04(-7.13-107.94) | 3.20(0.72-6.64) | 43.84(-7.93-102.99) | 53.20(-0.06-116.50) | 4.34(1.22-8.78) | 48.87(-2.49-110.56) | 0.48(0.37-0.58) | 1.17(1.07-1.27) | 0.42(0.32-0.53) |
| Low SDI | 86.32(-0.55-182.94) | 4.43(0.98-9.36) | 81.90(-2.27-174.93) | 73.80(7.64-159.91) | 5.32(1.38-10.65) | 68.48(4.54-151.40) | -0.64(-0.97--0.32) | 0.65(0.42-0.88) | -0.73(-1.06--0.39) |
| **GBD region** |  |  |  |  |  |  |  |  |  |
| Andean Latin America | 27.30(-56.68-120.44) | 3.91(0.67-8.14) | 23.40(-59.02-111.81) | 14.32(-30.92-62.62) | 4.11(0.80-8.19) | 10.21(-31.97-55.05) | -1.26(-1.83--0.68) | 0.56(0.35-0.78) | -1.75(-2.43--1.06) |
| Australasia | 89.71(8.28-206.88) | 18.64(6.52-38.84) | 71.07(-4.09-175.47) | 47.45(8.38-94.66) | 17.76(6.83-31.91) | 29.68(-0.29-66.11) | -1.70(-2.02--1.38) | -0.01(-0.07-0.06) | -2.46(-2.93--1.99) |
| Caribbean | 82.91(-4.17-202.51) | 4.86(1.54-9.32) | 78.05(-6.32-194.10) | 116.98(48.07-204.24) | 6.55(3.13-11.16) | 110.43(43.96-193.56) | 1.71(1.50-1.92) | 1.23(1.12-1.34) | 1.74(1.52-1.96) |
| Central Asia | 104.84(-11.03-265.40) | 13.14(2.40-29.55) | 91.70(-15.07-244.73) | 131.82(47.66-250.89) | 11.94(2.65-27.06) | 119.88(41.88-232.94) | 0.79(0.31-1.28) | -0.37(-0.41--0.34) | 0.92(0.39-1.46) |
| Central Europe | 293.22(61.81-611.46) | 22.26(4.79-46.63) | 270.96(52.51-568.72) | 219.26(105.05-365.08) | 20.43(5.79-40.14) | 198.83(96.88-328.16) | -0.99(-1.13--0.85) | -0.29(-0.33--0.25) | -1.05(-1.21--0.89) |
| Central Latin America | 32.75(-29.32-99.63) | 4.85(1.49-9.53) | 27.89(-30.88-91.63) | 9.74(-25.21-49.23) | 3.91(1.55-7.11) | 5.83(-28.07-43.90) | -5.02(-5.41--4.64) | -0.96(-1.11--0.80) | -6.39(-6.92--5.86) |
| Central Sub-Saharan Africa | 128.65(-25.24-306.21) | 7.38(1.31-16.93) | 121.26(-29.01-296.85) | 119.26(0.23-264.69) | 7.45(1.70-16.54) | 111.81(-3.10-253.04) | 0.30(-0.44-1.05) | 0.57(-0.16-1.31) | 0.28(-0.46-1.03) |
| East Asia | 244.44(52.33-458.03) | 12.15(2.62-24.12) | 232.29(49.32-438.97) | 158.57(29.13-307.50) | 16.03(3.27-32.90) | 142.53(25.65-275.92) | -1.31(-1.45--1.18) | 1.07(0.82-1.32) | -1.49(-1.62--1.37) |
| Eastern Europe | 464.45(277.21-747.24) | 23.45(7.31-47.37) | 440.99(268.81-701.15) | 590.80(456.40-764.36) | 22.30(7.79-45.75) | 568.50(445.45-728.40) | 0.23(-1.33-1.81) | -0.13(-0.41-0.16) | 0.25(-1.35-1.87) |
| Eastern Sub-Saharan Africa | 148.97(17.80-303.18) | 7.04(1.28-14.76) | 141.93(14.49-291.48) | 116.82(17.31-237.01) | 8.24(2.04-16.85) | 108.58(13.57-221.71) | -1.03(-1.31--0.75) | 0.51(0.30-0.72) | -1.13(-1.41--0.84) |
| High-income Asia Pacific | 151.92(12.89-303.59) | 24.58(4.72-50.76) | 127.34(4.33-264.56) | 45.13(-2.89-96.98) | 17.10(3.68-34.92) | 28.03(-9.59-66.25) | -4.38(-4.71--4.05) | -1.45(-1.61--1.30) | -5.45(-5.81--5.09) |
| High-income North America | 69.71(23.07-125.25) | 14.19(5.27-30.60) | 55.53(13.59-106.53) | 81.16(39.89-136.97) | 19.18(7.13-37.43) | 61.98(28.78-104.05) | 0.75(0.60-0.89) | 1.14(1.01-1.26) | 0.63(0.42-0.84) |
| North Africa and Middle East | 18.61(3.00-39.55) | 1.35(0.33-3.10) | 17.27(2.70-37.37) | 9.74(2.53-19.69) | 1.11(0.30-2.65) | 8.63(2.18-17.73) | -2.40(-2.60--2.20) | -0.82(-0.91--0.73) | -2.56(-2.77--2.34) |
| Oceania | 53.62(-10.43-139.03) | 3.70(0.73-8.10) | 49.92(-12.35-132.14) | 34.19(-14.40-98.86) | 3.28(0.59-7.30) | 30.91(-15.90-94.46) | -1.26(-1.74--0.78) | -0.19(-0.40-0.02) | -1.36(-1.87--0.85) |
| South Asia | 16.26(-25.71-62.02) | 2.01(0.39-4.50) | 14.25(-25.96-59.22) | 26.49(-14.51-75.58) | 3.31(0.97-7.04) | 23.18(-15.69-71.36) | 2.05(1.28-2.82) | 2.00(1.78-2.21) | 2.06(1.19-2.94) |
| Southeast Asia | 47.18(-12.39-110.96) | 4.12(0.76-8.68) | 43.06(-13.52-103.91) | 118.32(21.80-226.80) | 8.72(2.08-16.91) | 109.60(17.85-212.86) | 3.50(3.17-3.83) | 2.72(2.57-2.88) | 3.56(3.22-3.91) |
| Southern Latin America | 213.62(42.34-417.35) | 21.73(5.30-41.50) | 191.89(31.69-381.69) | 58.65(-5.21-131.50) | 12.16(2.97-24.94) | 46.48(-7.89-111.38) | -4.12(-4.27--3.97) | -1.89(-2.04--1.74) | -4.50(-4.64--4.35) |
| Southern Sub-Saharan Africa | 155.54(33.92-294.37) | 13.82(2.95-29.75) | 141.72(26.37-271.11) | 163.19(56.92-291.14) | 10.71(2.51-22.77) | 152.48(51.40-272.26) | -0.01(-0.44-0.42) | -1.12(-1.22--1.03) | 0.08(-0.39-0.54) |
| Tropical Latin America | 176.39(51.93-323.25) | 7.42(2.74-13.65) | 168.97(49.52-310.03) | 55.53(1.73-119.99) | 6.03(2.61-10.70) | 49.50(-0.70-109.26) | -4.13(-4.43--3.82) | -0.82(-1.02--0.61) | -4.37(-4.69--4.05) |
| Western Europe | 132.45(14.71-289.48) | 20.81(6.65-40.21) | 111.64(4.22-253.19) | 57.46(13.41-111.48) | 17.61(6.53-32.96) | 39.85(3.42-83.69) | -2.70(-2.79--2.60) | -0.53(-0.61--0.45) | -3.34(-3.46--3.23) |
| Western Sub-Saharan Africa | 167.65(31.48-326.71) | 9.97(2.60-20.54) | 157.68(27.75-307.49) | 150.12(38.21-280.26) | 11.42(3.11-22.75) | 138.70(34.23-258.04) | -0.56(-0.71--0.40) | 0.37(0.29-0.45) | -0.62(-0.78--0.46) |

**eTable 2:** ASMR, ASDR, Age-Standardized Rate of YLDs, and Age-Standardized Rate of YLLs of high alcohol use-related CVD in 2021 at 204 countries and regions worldwide. ASMR, age-standardized mortality rate; DALYs, disability-adjusted life years; ASDR, age-standardized rate of DALYs; YLDs, years lived with disability; YLLs, years of life lost; CVD, cardiovascular diseases.

| **Location Name** | **ASMR**  **per 100,000, N (95% UI)** | **ASDR**  **per 100,000, N (95% UI)** | **Age-Standardized Rate of YLDs**  **per 100,000, N (95% UI)** | **Age-Standardized Rate of YLLs**  **per 100,000, N (95% UI)** |
| --- | --- | --- | --- | --- |
| American Samoa | 0.05(-0.59-0.95) | -1.73(-25.06-24.68) | 1.03(-0.03-3.73) | -2.76(-25.79-22.25) |
| Antigua and Barbuda | 5.84(3.25-9.26) | 133.80(77.33-205.41) | 6.49(3.00-10.88) | 127.31(73.75-196.81) |
| Arab Republic of Egypt | 0.27(0.05-0.63) | 5.72(0.13-14.53) | 0.42(0.07-1.08) | 5.30(-0.01-13.70) |
| Argentine Republic | 2.69(0.23-5.87) | 53.56(-9.64-129.61) | 11.26(2.41-23.53) | 42.30(-15.31-109.18) |
| Australia | 2.00(0.45-4.23) | 47.32(9.52-92.76) | 18.27(7.04-32.76) | 29.05(0.04-63.50) |
| Barbados | 6.42(3.72-10.72) | 159.62(95.80-249.11) | 10.03(5.55-15.99) | 149.59(87.97-234.37) |
| Belize | 4.67(2.93-6.87) | 130.10(83.92-188.19) | 6.21(3.30-9.80) | 123.90(79.96-178.13) |
| Bermuda | 3.44(1.90-5.77) | 86.67(48.96-136.22) | 9.65(4.92-16.40) | 77.02(41.81-122.14) |
| Bolivarian Republic of Venezuela | 1.53(-0.27-4.04) | 25.11(-31.05-92.35) | 3.93(1.64-6.99) | 21.18(-35.03-87.37) |
| Bosnia and Herzegovina | 7.22(1.78-14.42) | 155.50(35.29-309.40) | 18.00(2.86-38.97) | 137.50(29.15-271.64) |
| Brunei Darussalam | 0.63(0.18-1.07) | 19.53(5.85-33.95) | 1.35(0.42-2.87) | 18.18(4.70-32.66) |
| Burkina Faso | 9.54(3.42-17.04) | 208.57(75.67-375.90) | 11.49(4.12-21.70) | 197.09(70.56-356.39) |
| Canada | 1.36(0.39-2.90) | 43.12(13.57-83.04) | 19.32(7.25-38.36) | 23.80(4.01-48.97) |
| Central African Republic | 4.99(-0.49-12.93) | 101.72(-37.94-284.47) | 4.73(0.85-11.48) | 96.99(-40.60-276.37) |
| Commonwealth of Dominica | 7.07(3.64-11.85) | 169.78(87.96-279.95) | 6.10(2.62-10.44) | 163.67(84.01-271.27) |
| Commonwealth of the Bahamas | 8.51(5.37-12.61) | 243.50(159.03-349.90) | 6.67(3.22-11.78) | 236.84(155.60-340.54) |
| Cook Islands | 9.81(5.48-15.63) | 257.57(133.76-416.94) | 22.73(6.99-44.44) | 234.84(120.69-383.04) |
| Czech Republic | 3.00(0.46-7.44) | 70.69(5.94-162.81) | 25.60(6.67-51.31) | 45.09(-5.97-121.90) |
| Democratic People's Republic of Korea | 6.76(0.81-13.87) | 178.06(6.40-369.52) | 12.66(2.41-26.04) | 165.41(2.12-352.82) |
| Democratic Republic of Sao Tome and Principe | 7.47(0.93-14.82) | 172.58(23.52-341.28) | 17.53(3.96-35.68) | 155.06(19.21-308.46) |
| Democratic Republic of the Congo | 4.58(0.01-10.58) | 91.03(-13.87-227.14) | 5.61(0.89-13.23) | 85.42(-15.60-216.24) |
| Democratic Republic of Timor-Leste | 5.05(0.86-10.19) | 121.75(14.76-250.68) | 7.45(1.88-14.87) | 114.30(9.74-242.26) |
| Democratic Socialist Republic of Sri Lanka | 2.83(0.69-6.12) | 61.39(12.92-128.43) | 6.29(1.61-13.19) | 55.10(11.02-115.73) |
| Dominican Republic | 2.42(-0.26-6.38) | 55.60(-26.12-163.60) | 6.02(1.85-11.30) | 49.58(-29.98-155.19) |
| Eastern Republic of Uruguay | 4.14(1.22-8.20) | 84.54(18.16-166.48) | 12.62(2.95-26.02) | 71.92(10.72-143.17) |
| Federal Democratic Republic of Ethiopia | 3.45(0.43-7.26) | 76.75(3.22-165.61) | 6.44(1.87-13.12) | 70.32(-0.77-155.79) |
| Federal Democratic Republic of Nepal | 1.36(-0.64-4.33) | 26.17(-24.76-92.79) | 3.26(0.50-7.47) | 22.91(-25.81-84.86) |
| Federal Republic of Germany | 4.65(2.30-7.93) | 103.85(45.76-178.63) | 28.92(11.21-52.52) | 74.93(29.53-128.17) |
| Federal Republic of Nigeria | 6.51(1.44-12.63) | 137.69(34.41-266.42) | 12.70(3.79-24.92) | 124.99(29.98-242.71) |
| Federal Republic of Somalia | 0.00(0.00-0.01) | 0.27(0.14-0.62) | 0.20(0.12-0.31) | 0.07(0.01-0.41) |
| Federated States of Micronesia | 2.28(-1.61-7.93) | 58.69(-64.41-232.65) | 6.66(1.19-15.26) | 52.03(-68.72-223.26) |
| Federative Republic of Brazil | 2.48(0.49-5.01) | 54.83(1.91-117.51) | 5.99(2.62-10.62) | 48.84(-0.51-108.38) |
| French Republic | 2.35(0.69-4.59) | 50.51(12.56-94.54) | 17.51(6.36-32.28) | 32.99(3.92-68.67) |
| Gabonese Republic | 11.72(3.70-21.07) | 240.13(64.44-457.77) | 16.24(3.94-34.03) | 223.88(55.24-429.95) |
| Georgia | 10.08(3.14-20.75) | 213.84(57.24-425.31) | 14.99(3.52-32.11) | 198.85(51.64-394.57) |
| Grand Duchy of Luxembourg | 2.74(0.67-5.49) | 50.32(9.71-100.37) | 13.01(5.02-23.37) | 37.30(0.73-81.02) |
| Greenland | 4.84(1.26-9.82) | 118.59(34.44-226.57) | 18.93(5.83-39.40) | 99.65(25.83-198.73) |
| Grenada | 8.62(5.47-12.58) | 225.20(149.21-316.68) | 8.35(4.25-14.16) | 216.85(144.39-305.90) |
| Guam | 0.14(-1.93-2.67) | 6.07(-66.71-91.94) | 9.05(0.73-23.42) | -2.98(-72.44-76.38) |
| Hashemite Kingdom of Jordan | 0.22(0.10-0.44) | 5.02(1.97-9.86) | 0.68(0.17-1.72) | 4.34(1.78-8.37) |
| Hellenic Republic | 2.45(-0.82-6.58) | 27.28(-47.39-113.73) | 12.01(2.75-24.59) | 15.27(-54.12-91.36) |
| Hungary | 12.65(8.91-18.22) | 340.79(242.64-471.88) | 21.34(6.72-40.85) | 319.45(231.22-440.18) |
| Independent State of Papua New Guinea | 1.44(-0.08-3.66) | 35.45(-5.17-98.15) | 2.20(0.43-5.02) | 33.26(-7.21-94.13) |
| Independent State of Samoa | 1.68(-0.62-5.06) | 43.91(-25.23-139.90) | 5.46(1.03-12.17) | 38.45(-27.14-130.37) |
| Ireland | 1.02(-0.35-3.06) | 18.95(-12.29-59.75) | 10.12(3.28-19.87) | 8.83(-19.81-44.03) |
| Islamic Republic of Afghanistan | 0.14(0.02-0.36) | 3.21(-0.31-9.13) | 0.14(0.03-0.39) | 3.07(-0.42-8.85) |
| Islamic Republic of Iran | 0.37(0.14-0.75) | 8.95(3.12-18.04) | 1.06(0.32-2.42) | 7.89(2.74-15.65) |
| Islamic Republic of Mauritania | 0.05(0.01-0.12) | 1.55(0.43-3.71) | 0.26(0.16-0.40) | 1.29(0.17-3.52) |
| Islamic Republic of Pakistan | 0.13(-0.53-0.89) | -0.38(-21.01-20.88) | 1.17(0.08-3.02) | -1.55(-21.68-19.38) |
| Jamaica | 4.98(2.49-8.58) | 122.79(63.95-203.86) | 5.16(2.43-8.56) | 117.63(61.01-196.44) |
| Japan | 1.52(-0.28-3.55) | 41.15(-9.02-92.99) | 16.53(2.97-34.42) | 24.61(-14.48-66.36) |
| Kingdom of Bahrain | 0.50(0.23-0.86) | 10.36(4.08-18.53) | 1.04(0.41-2.12) | 9.31(3.60-16.80) |
| Kingdom of Belgium | 2.11(0.25-4.75) | 44.14(1.23-97.49) | 13.64(3.89-26.74) | 30.50(-4.68-74.98) |
| Kingdom of Bhutan | 0.16(-0.38-0.80) | 1.48(-13.79-17.73) | 0.85(-0.01-2.36) | 0.64(-14.01-15.90) |
| Kingdom of Cambodia | 9.18(2.82-16.68) | 216.13(58.11-398.50) | 13.09(3.66-25.79) | 203.04(53.47-377.91) |
| Kingdom of Denmark | 2.53(0.12-5.87) | 48.01(-1.65-108.11) | 15.46(4.87-29.71) | 32.54(-8.77-84.33) |
| Kingdom of Eswatini | 8.87(3.27-15.71) | 199.94(65.17-364.31) | 7.99(2.04-16.83) | 191.95(60.85-349.40) |
| Kingdom of Lesotho | 10.41(3.59-19.57) | 256.63(73.15-503.58) | 7.11(1.53-15.84) | 249.52(69.25-490.87) |
| Kingdom of Morocco | 0.18(0.06-0.33) | 4.64(1.30-8.90) | 0.41(0.12-0.95) | 4.23(1.06-8.31) |
| Kingdom of Norway | 0.88(-0.52-2.84) | 21.64(-11.26-62.27) | 13.61(2.47-29.29) | 8.03(-16.10-38.29) |
| Kingdom of Saudi Arabia | 0.19(0.04-0.44) | 4.36(0.11-10.70) | 0.27(0.05-0.75) | 4.08(0.04-10.28) |
| Kingdom of Spain | 1.88(0.41-3.73) | 38.77(3.00-79.48) | 15.12(5.21-28.70) | 23.65(-5.58-57.01) |
| Kingdom of Sweden | 1.87(0.12-4.43) | 45.67(5.87-94.12) | 20.20(8.18-37.43) | 25.47(-4.16-65.56) |
| Kingdom of Thailand | 2.57(0.36-5.30) | 76.79(7.03-155.81) | 9.96(2.20-19.78) | 66.83(2.81-145.73) |
| Kingdom of the Netherlands | 2.76(0.48-5.97) | 52.14(8.00-109.46) | 15.21(4.91-29.66) | 36.92(3.51-81.39) |
| Kingdom of Tonga | 0.17(-0.47-1.04) | 3.11(-15.65-28.82) | 1.81(0.15-4.59) | 1.30(-16.32-25.14) |
| Kyrgyz Republic | 10.97(8.14-14.71) | 368.50(277.59-484.98) | 12.10(5.23-22.97) | 356.40(269.15-465.34) |
| Lao People's Democratic Republic | 10.57(0.91-22.21) | 258.39(2.16-557.49) | 19.37(4.41-40.69) | 239.02(-8.18-521.55) |
| Lebanese Republic | 0.45(-0.01-1.17) | 10.13(-2.43-29.26) | 2.69(0.34-6.87) | 7.44(-3.01-22.52) |
| Malaysia | 0.46(-0.47-1.70) | 12.00(-14.56-46.53) | 3.43(0.34-8.40) | 8.57(-15.80-40.54) |
| Mongolia | 10.53(3.65-18.87) | 313.59(115.44-538.16) | 18.15(5.93-37.23) | 295.43(106.54-520.43) |
| Montenegro | 16.75(5.53-31.66) | 322.04(99.88-588.99) | 14.99(4.61-29.43) | 307.05(90.16-572.16) |
| New Zealand | 2.35(0.34-5.29) | 48.37(4.16-105.10) | 15.14(5.32-29.51) | 33.22(-3.96-81.41) |
| North Macedonia | 19.22(4.85-38.46) | 333.35(86.66-631.84) | 19.89(4.12-40.80) | 313.46(82.27-594.20) |
| Northern Mariana Islands | 1.57(-0.99-5.30) | 38.54(-32.83-143.27) | 7.23(0.25-18.32) | 31.31(-36.22-132.23) |
| Palestine | 0.71(0.29-1.35) | 15.48(5.35-30.47) | 1.09(0.33-2.49) | 14.40(5.14-28.14) |
| People's Democratic Republic of Algeria | 0.61(0.26-1.12) | 12.30(4.79-22.45) | 1.25(0.39-2.91) | 11.05(4.35-20.31) |
| People's Republic of Bangladesh | 0.39(0.04-0.96) | 9.57(0.34-24.69) | 0.57(0.07-1.48) | 9.00(0.17-23.41) |
| People's Republic of China | 7.56(1.83-14.65) | 160.48(29.51-312.69) | 16.17(3.28-33.19) | 144.32(26.16-280.29) |
| Plurinational State of Bolivia | 1.94(-0.64-5.38) | 33.51(-33.54-112.86) | 3.84(0.91-7.76) | 29.67(-34.85-107.24) |
| Portuguese Republic | 4.08(1.05-7.95) | 71.43(14.02-139.41) | 11.71(4.71-21.07) | 59.72(7.22-121.47) |
| Principality of Andorra | 2.11(0.46-4.40) | 44.38(7.36-88.79) | 14.03(5.65-25.64) | 30.35(0.15-68.73) |
| Principality of Monaco | 3.84(0.76-9.23) | 78.29(16.24-171.82) | 12.27(1.68-30.69) | 66.02(12.52-146.36) |
| Puerto Rico | 1.22(0.59-2.12) | 33.04(12.94-56.46) | 4.82(2.33-8.00) | 28.22(10.01-50.17) |
| Republic of Albania | 5.68(0.67-13.10) | 93.29(-1.78-220.03) | 8.87(2.10-17.83) | 84.42(-6.56-202.45) |
| Republic of Angola | 7.89(2.13-14.93) | 174.35(30.43-343.14) | 10.83(2.90-23.34) | 163.53(24.81-322.88) |
| Republic of Armenia | 2.32(0.80-5.00) | 56.95(9.99-132.57) | 9.92(2.47-22.04) | 47.03(5.88-114.00) |
| Republic of Austria | 2.47(0.81-4.98) | 61.34(19.83-118.83) | 24.19(8.86-45.98) | 37.16(6.10-78.27) |
| Republic of Azerbaijan | 4.28(1.40-8.42) | 91.11(20.57-183.20) | 9.65(2.54-21.15) | 81.46(14.78-170.73) |
| Republic of Belarus | 8.71(4.17-16.60) | 293.88(149.73-496.78) | 24.63(6.80-50.57) | 269.25(134.70-450.03) |
| Republic of Benin | 4.19(0.78-9.33) | 100.38(17.71-218.45) | 6.85(1.59-14.80) | 93.54(15.98-201.35) |
| Republic of Botswana | 3.91(1.39-7.30) | 91.12(26.04-171.56) | 7.42(1.38-16.62) | 83.70(22.92-161.05) |
| Republic of Bulgaria | 26.42(11.03-45.13) | 500.74(188.86-859.90) | 28.13(6.12-57.42) | 472.61(181.42-812.00) |
| Republic of Burundi | 7.22(1.27-15.29) | 166.87(17.65-350.74) | 9.92(2.63-19.68) | 156.94(12.95-331.40) |
| Republic of Cabo Verde | 5.17(-0.96-12.13) | 119.66(-19.49-270.95) | 12.51(3.10-25.23) | 107.15(-23.66-247.38) |
| Republic of Cameroon | 12.54(2.99-23.68) | 288.57(62.16-547.10) | 18.20(4.91-35.31) | 270.38(56.94-516.13) |
| Republic of Chad | 6.44(0.31-16.71) | 153.88(5.23-401.52) | 8.77(0.76-21.03) | 145.11(2.88-380.94) |
| Republic of Chile | 3.49(0.91-6.67) | 65.08(4.14-132.94) | 14.08(4.00-27.50) | 51.00(-0.59-108.72) |
| Republic of Colombia | 0.26(-0.35-1.14) | 3.70(-15.57-26.05) | 2.59(0.95-5.00) | 1.11(-16.93-22.44) |
| Republic of Costa Rica | 1.01(0.04-2.48) | 17.95(-9.01-51.08) | 4.57(1.90-8.28) | 13.38(-12.10-44.72) |
| Republic of Croatia | 6.97(3.45-12.63) | 148.75(68.40-257.84) | 16.91(2.42-35.34) | 131.84(62.58-222.02) |
| Republic of Cuba | 5.09(3.13-7.89) | 137.02(87.35-199.49) | 8.02(4.35-12.82) | 129.00(82.15-189.52) |
| Republic of Cyprus | 2.24(-0.29-5.67) | 22.70(-25.97-77.15) | 6.77(2.05-12.96) | 15.94(-28.93-67.44) |
| Republic of C么te d'Ivoire | 11.71(2.22-23.26) | 263.64(50.89-517.84) | 17.81(4.45-36.14) | 245.83(43.54-486.50) |
| Republic of Djibouti | 0.07(-0.33-0.47) | -0.86(-13.79-9.98) | 0.37(-0.37-1.34) | -1.23(-13.63-8.84) |
| Republic of Ecuador | 0.33(-0.68-1.47) | -0.28(-34.96-34.13) | 2.85(0.64-5.77) | -3.13(-36.69-29.10) |
| Republic of El Salvador | 0.11(-0.79-1.21) | -5.60(-36.63-25.40) | 2.14(0.72-4.17) | -7.74(-38.22-22.14) |
| Republic of Equatorial Guinea | 7.94(1.60-16.36) | 156.98(16.31-332.04) | 12.71(3.38-27.06) | 144.27(11.10-312.85) |
| Republic of Estonia | 10.91(7.13-16.54) | 280.65(194.29-390.55) | 19.42(6.75-39.31) | 261.23(187.74-361.74) |
| Republic of Fiji | 1.10(-1.51-4.33) | 22.45(-57.26-115.83) | 6.35(1.00-14.05) | 16.10(-61.05-106.45) |
| Republic of Finland | 3.30(1.37-6.08) | 94.22(44.30-156.04) | 18.89(6.60-35.40) | 75.33(34.45-124.86) |
| Republic of Ghana | 10.78(2.14-22.04) | 258.44(55.08-525.82) | 16.96(3.07-36.85) | 241.48(50.22-493.78) |
| Republic of Guatemala | 0.37(-0.40-1.50) | 2.91(-20.30-31.98) | 1.96(0.66-3.69) | 0.95(-21.49-29.04) |
| Republic of Guinea | 2.94(0.05-7.13) | 67.91(0.11-160.19) | 4.65(0.87-10.48) | 63.25(-1.96-150.23) |
| Republic of Guinea-Bissau | 9.73(1.88-19.16) | 238.32(39.77-478.53) | 10.60(2.29-22.46) | 227.71(36.56-462.57) |
| Republic of Guyana | 10.91(5.80-18.17) | 289.56(154.04-471.60) | 7.20(2.89-12.52) | 282.36(150.97-461.79) |
| Republic of Haiti | 8.77(2.58-18.63) | 207.37(54.92-433.54) | 6.23(2.58-11.15) | 201.14(50.67-423.84) |
| Republic of Honduras | 3.19(1.12-6.14) | 67.66(21.16-130.51) | 3.06(1.43-5.40) | 64.59(19.13-127.46) |
| Republic of Iceland | 0.46(-1.01-2.75) | 7.65(-28.87-56.22) | 13.14(3.24-26.00) | -5.49(-36.54-34.13) |
| Republic of India | 1.44(-0.35-3.70) | 31.24(-15.86-87.90) | 3.85(1.16-8.08) | 27.39(-17.41-82.36) |
| Republic of Indonesia | 0.59(0.02-1.46) | 14.70(-0.67-37.46) | 1.14(0.18-2.90) | 13.56(-0.78-34.72) |
| Republic of Iraq | 0.32(0.11-0.67) | 7.08(2.17-14.83) | 0.41(0.11-0.98) | 6.67(2.05-13.99) |
| Republic of Italy | 3.60(1.50-6.42) | 57.15(14.18-109.12) | 13.07(5.59-23.30) | 44.08(7.91-89.14) |
| Republic of Kazakhstan | 6.97(1.97-15.10) | 178.50(52.34-369.46) | 16.96(2.54-40.84) | 161.54(44.60-328.06) |
| Republic of Kenya | 5.49(1.25-10.55) | 121.08(17.71-238.39) | 9.03(1.99-19.37) | 112.06(13.32-224.26) |
| Republic of Kiribati | 0.82(-0.55-3.54) | 20.86(-26.16-109.63) | 3.09(0.05-9.32) | 17.76(-27.60-102.53) |
| Republic of Korea | 2.71(0.50-5.77) | 63.90(11.39-132.30) | 19.56(5.19-39.38) | 44.34(4.62-94.64) |
| Republic of Latvia | 23.30(16.44-33.81) | 747.48(569.51-974.32) | 29.09(8.78-58.97) | 718.39(554.23-926.01) |
| Republic of Liberia | 7.28(1.78-14.17) | 174.67(42.97-341.47) | 9.97(2.46-20.76) | 164.70(39.84-324.63) |
| Republic of Lithuania | 7.84(4.44-13.31) | 222.18(127.10-351.56) | 23.61(6.13-50.82) | 198.57(115.67-313.92) |
| Republic of Madagascar | 4.14(0.20-9.53) | 93.34(-12.43-236.93) | 5.21(0.56-12.35) | 88.13(-13.67-227.36) |
| Republic of Malawi | 4.91(1.13-9.22) | 114.73(11.54-226.67) | 6.12(1.45-13.05) | 108.61(10.62-217.73) |
| Republic of Maldives | 0.31(-0.21-1.13) | 6.13(-9.63-28.22) | 1.93(0.08-5.13) | 4.20(-10.21-23.34) |
| Republic of Mali | 2.10(0.58-4.28) | 45.64(12.62-90.02) | 3.19(1.18-6.31) | 42.44(11.38-84.06) |
| Republic of Malta | 0.35(-1.10-2.65) | 2.07(-34.94-44.32) | 7.05(1.92-14.38) | -4.98(-38.75-33.78) |
| Republic of Mauritius | 2.23(-0.44-5.55) | 53.95(-24.25-142.33) | 8.47(1.76-17.43) | 45.48(-28.40-125.86) |
| Republic of Moldova | 11.24(6.26-18.39) | 290.73(152.20-467.35) | 22.39(6.53-47.10) | 268.35(137.32-427.21) |
| Republic of Mozambique | 6.65(1.43-14.18) | 164.55(26.25-359.13) | 5.20(0.03-12.71) | 159.35(25.13-348.11) |
| Republic of Namibia | 15.42(5.60-27.69) | 307.78(96.93-554.86) | 16.58(3.51-36.01) | 291.19(89.84-532.80) |
| Republic of Nauru | 5.99(-2.76-17.46) | 154.95(-127.66-493.60) | 16.04(2.64-34.46) | 138.91(-138.02-457.46) |
| Republic of Nicaragua | 0.81(-0.08-2.06) | 15.53(-11.27-48.39) | 3.47(1.26-6.42) | 12.06(-13.91-42.03) |
| Republic of Niue | 3.69(-1.00-9.84) | 97.89(-40.52-273.92) | 12.99(1.75-27.90) | 84.90(-47.48-257.92) |
| Republic of Palau | 1.95(-3.23-9.53) | 46.60(-114.68-265.91) | 12.55(0.81-30.27) | 34.05(-121.43-244.89) |
| Republic of Panama | 1.94(0.46-3.95) | 39.97(4.46-82.90) | 5.00(2.28-8.94) | 34.97(1.39-74.66) |
| Republic of Paraguay | 4.60(0.54-9.74) | 88.82(-7.81-204.66) | 7.91(2.29-14.80) | 80.91(-10.15-193.63) |
| Republic of Peru | 0.97(-0.58-2.83) | 16.38(-29.52-64.81) | 4.81(0.80-9.46) | 11.57(-31.01-57.18) |
| Republic of Poland | 6.60(3.60-11.61) | 172.44(94.99-280.89) | 18.86(7.46-34.71) | 153.58(85.08-245.66) |
| Republic of Rwanda | 7.83(1.65-15.27) | 177.30(24.45-362.65) | 11.06(2.72-22.70) | 166.24(21.12-344.10) |
| Republic of San Marino | 2.32(0.20-5.39) | 49.05(4.79-110.55) | 14.28(0.68-31.24) | 34.77(3.70-81.29) |
| Republic of Senegal | 0.76(-0.20-2.27) | 15.57(-7.92-48.66) | 1.32(-0.00-3.62) | 14.26(-7.87-45.91) |
| Republic of Serbia | 12.58(4.57-23.79) | 251.69(95.24-449.90) | 15.69(2.84-33.64) | 236.01(92.83-421.18) |
| Republic of Seychelles | 3.94(1.55-6.88) | 104.95(34.86-187.50) | 9.88(2.65-20.46) | 95.07(30.33-170.34) |
| Republic of Sierra Leone | 5.80(1.24-11.60) | 140.53(31.97-273.03) | 9.45(2.52-18.96) | 131.08(29.16-256.18) |
| Republic of Singapore | -0.20(-0.70-0.30) | -3.79(-20.39-13.12) | 4.07(0.57-9.16) | -7.87(-23.41-6.10) |
| Republic of Slovenia | 3.63(1.60-6.76) | 72.37(33.58-130.92) | 9.97(2.78-22.52) | 62.40(29.40-108.57) |
| Republic of South Africa | 7.36(3.16-12.55) | 160.38(57.89-280.24) | 11.29(2.74-24.13) | 149.09(53.61-262.77) |
| Republic of South Sudan | 0.12(-0.27-0.71) | 0.55(-13.08-15.22) | 0.33(-0.28-1.09) | 0.23(-12.80-14.47) |
| Republic of Sudan | 0.07(0.02-0.16) | 1.72(0.34-3.98) | 0.04(0.01-0.06) | 1.68(0.31-3.94) |
| Republic of Suriname | 4.09(1.07-8.19) | 103.69(22.07-213.86) | 5.02(1.72-9.60) | 98.66(18.15-205.89) |
| Republic of Tajikistan | 0.97(0.28-2.00) | 29.15(4.61-62.52) | 3.08(0.77-6.86) | 26.07(2.79-55.15) |
| Republic of the Congo | 10.92(1.76-22.89) | 215.66(6.27-479.43) | 14.93(2.88-32.70) | 200.72(-0.89-454.56) |
| Republic of the Gambia | 6.97(1.21-15.79) | 160.61(24.60-354.09) | 9.44(1.70-20.20) | 151.16(20.23-334.32) |
| Republic of the Marshall Islands | 3.16(-0.34-8.29) | 82.81(-22.31-231.70) | 6.09(1.33-13.71) | 76.73(-29.55-220.26) |
| Republic of the Niger | 0.55(0.05-1.64) | 11.64(-1.15-36.46) | 0.79(0.05-2.53) | 10.85(-1.16-34.13) |
| Republic of the Philippines | 7.39(1.17-15.05) | 189.77(7.07-392.26) | 16.77(4.10-32.87) | 173.00(-4.63-368.02) |
| Republic of the Union of Myanmar | 6.15(1.51-11.88) | 161.57(35.70-318.18) | 8.18(2.21-16.24) | 153.39(31.59-302.58) |
| Republic of Trinidad and Tobago | 3.64(0.92-7.67) | 82.96(11.44-177.78) | 6.35(2.10-11.98) | 76.61(9.66-167.33) |
| Republic of Tunisia | 0.92(0.38-1.75) | 20.93(7.39-39.27) | 1.95(0.61-4.32) | 18.97(6.45-35.17) |
| Republic of Turkey | 0.76(0.19-1.61) | 16.04(1.68-35.91) | 2.40(0.58-5.68) | 13.64(0.97-31.76) |
| Republic of Uganda | 6.61(1.37-13.41) | 157.85(24.30-330.27) | 12.62(3.59-25.58) | 145.23(15.97-309.07) |
| Republic of Uzbekistan | 2.39(0.31-5.57) | 56.23(-5.99-146.96) | 9.97(1.87-22.74) | 46.26(-9.70-127.77) |
| Republic of Vanuatu | 2.27(-1.15-6.79) | 59.03(-48.81-192.87) | 7.30(1.45-16.10) | 51.73(-53.44-182.80) |
| Republic of Yemen | 0.41(0.08-0.94) | 9.75(1.01-23.21) | 0.52(0.13-1.23) | 9.23(0.76-22.29) |
| Republic of Zambia | 10.39(3.23-20.25) | 228.27(55.16-473.27) | 11.62(2.80-24.42) | 216.64(49.35-456.33) |
| Republic of Zimbabwe | 6.79(1.29-12.88) | 149.49(19.40-300.99) | 6.78(0.84-14.92) | 142.72(15.57-290.82) |
| Romania | 13.93(5.30-25.74) | 286.71(107.21-507.53) | 21.61(3.56-45.53) | 265.11(98.18-471.13) |
| Russian Federation | 18.57(13.62-25.63) | 640.52(500.00-815.84) | 22.06(7.98-44.05) | 618.47(485.13-778.63) |
| Saint Kitts and Nevis | 5.29(1.94-12.79) | 133.30(56.08-291.13) | 4.61(1.03-12.49) | 128.69(54.31-278.37) |
| Saint Lucia | 11.69(7.36-17.34) | 298.87(195.60-419.13) | 11.63(6.34-18.43) | 287.25(186.98-404.83) |
| Saint Vincent and the Grenadines | 11.30(6.44-17.57) | 269.21(160.08-409.22) | 10.25(5.28-16.83) | 258.96(152.06-393.33) |
| Slovak Republic | 4.61(0.83-10.80) | 118.77(18.40-252.68) | 26.27(4.46-54.36) | 92.50(3.08-209.38) |
| Socialist Republic of Viet Nam | 16.03(3.93-28.73) | 373.55(80.21-688.37) | 20.70(4.42-39.32) | 352.85(77.38-650.05) |
| Solomon Islands | 0.65(-1.35-3.49) | 14.05(-48.46-99.12) | 3.29(0.42-7.73) | 10.75(-50.51-93.80) |
| State of Eritrea | 1.28(-0.55-3.95) | 26.17(-28.48-95.23) | 2.05(-0.06-5.47) | 24.13(-29.00-90.87) |
| State of Israel | 0.18(-0.38-1.00) | 5.13(-10.53-25.11) | 4.97(0.68-11.40) | 0.17(-12.87-14.83) |
| State of Kuwait | 0.12(0.07-0.18) | 2.97(1.57-4.65) | 0.22(0.08-0.50) | 2.76(1.36-4.29) |
| State of Libya | 0.27(0.08-0.54) | 6.27(1.32-13.51) | 0.55(0.16-1.27) | 5.71(1.04-12.31) |
| State of Qatar | 0.17(0.04-0.38) | 4.16(0.59-9.60) | 0.94(0.24-2.23) | 3.22(0.23-7.46) |
| Sultanate of Oman | 0.19(0.02-0.47) | 4.49(0.07-12.00) | 0.66(0.10-1.76) | 3.83(-0.27-10.33) |
| Swiss Confederation | 1.93(0.60-4.05) | 37.74(9.74-77.10) | 11.76(3.49-23.29) | 25.98(2.77-56.31) |
| Syrian Arab Republic | 0.41(-0.03-1.10) | 8.62(-2.79-24.37) | 0.93(0.20-2.24) | 7.69(-3.20-23.31) |
| Taiwan (Province of China) | 1.77(0.64-3.19) | 54.74(19.59-96.63) | 11.40(3.21-23.07) | 43.34(13.88-77.83) |
| Togolese Republic | 5.15(0.78-11.35) | 125.33(17.72-269.29) | 7.61(1.52-17.64) | 117.73(15.11-254.23) |
| Tokelau | 2.10(-1.14-6.71) | 50.00(-44.03-176.93) | 6.92(1.32-15.40) | 43.09(-47.81-161.34) |
| Turkmenistan | 6.76(1.56-14.50) | 181.90(23.88-387.27) | 15.04(2.56-34.63) | 166.86(18.84-359.64) |
| Tuvalu | 1.27(-1.98-5.83) | 27.49(-73.75-167.21) | 5.58(0.89-12.69) | 21.91(-77.65-155.19) |
| Ukraine | 15.63(10.25-22.90) | 544.00(365.47-785.98) | 22.06(7.07-45.94) | 521.94(345.86-749.15) |
| Union of the Comoros | 0.69(0.09-1.71) | 14.78(1.06-38.12) | 1.30(0.32-2.99) | 13.47(0.65-35.62) |
| United Arab Emirates | 1.05(0.11-2.71) | 22.43(1.38-59.28) | 4.92(0.57-13.01) | 17.51(-0.27-45.90) |
| United Kingdom of Great Britain and Northern Ireland | 1.42(-0.43-4.03) | 30.41(-11.91-88.12) | 13.40(3.79-26.67) | 17.01(-19.56-62.16) |
| United Mexican States | 0.67(-0.45-2.06) | 5.66(-32.81-45.67) | 4.74(1.96-8.80) | 0.92(-36.12-38.21) |
| United Republic of Tanzania | 7.16(1.95-13.47) | 146.70(27.76-285.05) | 12.98(3.34-27.15) | 133.72(22.05-265.66) |
| United States of America | 3.03(1.53-5.43) | 85.72(43.17-143.16) | 19.16(7.16-37.38) | 66.56(31.76-111.21) |
| United States Virgin Islands | 2.70(-0.07-6.66) | 64.89(-7.17-161.07) | 7.12(1.07-16.01) | 57.77(-8.59-148.99) |

**eTable 3:** ASMR, ASDR, Age-Standardized Rate of YLDs, and Age-Standardized Rate of YLLs of high alcohol use-related CVD in males and females across 21 GBD regions in 2021. ASMR, age-standardized mortality rate; DALYs, disability-adjusted life years; ASDR, age-standardized rate of DALYs; YLDs, years lived with disability; YLLs, years of life lost; CVD, cardiovascular diseases.

| **Location Name** | **ASMR**  **per 100,000, N (95% UI)** | | **ASDR**  **per 100,000, N (95% UI)** | | **Age-Standardized Rate of YLDs**  **per 100,000, N (95% UI)** | | **Age-Standardized Rate of YLLs**  **per 100,000, N (95% UI)** | |
| --- | --- | --- | --- | --- | --- | --- | --- | --- |
|  | **Male** | **Female** | **Male** | **Female** | **Male** | **Female** | **Male** | **Female** |
| Andean Latin America | 1.59(-1.03-4.63) | 0.36(-0.15-1.16) | 22.58(-56.18-101.32) | 7.93(-6.82-27.89) | 6.74(1.50-12.79) | 1.77(0.19-4.18) | 15.85(-58.31-90.43) | 6.16(-7.34-24.88) |
| Australasia | 3.17(1.16-6.13) | 1.13(-0.12-2.97) | 79.51(25.85-145.88) | 18.92(-6.94-50.91) | 28.65(11.65-50.67) | 7.94(2.20-16.30) | 50.86(9.54-102.63) | 10.98(-9.28-35.76) |
| Caribbean | 8.54(4.13-14.40) | 1.06(0.26-2.21) | 212.83(93.60-361.19) | 31.28(6.73-64.31) | 11.27(5.44-18.40) | 2.41(0.82-4.91) | 201.57(86.95-344.23) | 28.87(5.20-59.53) |
| Central Asia | 11.38(5.00-21.59) | 1.21(0.37-2.90) | 264.69(97.19-497.77) | 32.87(11.22-69.52) | 21.33(5.33-46.66) | 4.46(0.45-11.46) | 243.36(87.50-460.49) | 28.41(9.94-60.53) |
| Central Europe | 19.73(10.19-33.27) | 3.18(1.04-6.64) | 423.33(219.64-678.48) | 62.44(17.50-128.07) | 36.07(11.50-68.05) | 8.39(1.01-20.61) | 387.26(206.89-613.25) | 54.05(15.81-110.53) |
| Central Latin America | 1.54(-0.59-4.22) | 0.11(-0.12-0.48) | 20.32(-47.48-94.48) | 2.17(-4.83-11.57) | 7.27(3.20-12.99) | 1.09(0.20-2.50) | 13.05(-52.59-84.76) | 1.08(-5.39-9.46) |
| Central Sub-Saharan Africa | 8.18(0.26-18.41) | 4.19(1.16-7.88) | 164.55(-25.42-402.16) | 86.60(19.24-165.23) | 10.77(2.87-22.37) | 4.92(0.74-12.04) | 153.79(-30.58-383.38) | 81.68(18.56-155.93) |
| East Asia | 16.00(3.97-31.27) | 0.97(0.21-2.06) | 320.49(60.63-623.04) | 19.77(4.27-41.09) | 30.34(6.18-61.70) | 2.92(0.42-6.86) | 290.15(54.70-561.05) | 16.86(3.18-34.93) |
| Eastern Europe | 32.62(23.03-46.10) | 6.91(5.16-10.32) | 1036.87(783.77-1360.19) | 245.08(191.19-328.77) | 36.96(13.48-73.84) | 12.20(3.98-27.38) | 999.91(765.29-1291.85) | 232.89(185.44-305.76) |
| Eastern Sub-Saharan Africa | 8.34(1.48-16.71) | 2.66(0.95-4.63) | 184.96(17.45-382.89) | 56.54(18.48-103.89) | 12.63(3.46-25.12) | 4.39(0.76-10.01) | 172.32(11.56-357.87) | 52.15(16.11-94.70) |
| High-income Asia Pacific | 2.94(-0.19-6.53) | 0.78(0.01-1.78) | 76.35(-4.03-160.17) | 18.76(-2.09-43.22) | 27.28(6.00-54.62) | 8.11(1.49-18.41) | 49.07(-16.14-114.90) | 10.65(-3.84-26.70) |
| High-income North America | 4.55(2.42-7.46) | 1.45(0.37-3.09) | 128.42(72.92-205.20) | 39.30(10.18-77.26) | 26.77(11.26-50.07) | 12.62(3.24-26.51) | 101.65(55.05-158.03) | 26.68(4.93-55.81) |
| North Africa and Middle East | 0.78(0.25-1.51) | 0.12(0.05-0.27) | 16.91(4.17-33.88) | 2.45(0.95-5.32) | 1.94(0.53-4.62) | 0.24(0.05-0.59) | 14.96(3.47-30.43) | 2.21(0.87-4.77) |
| Oceania | 2.45(-0.70-6.68) | 0.27(0.00-0.72) | 59.08(-28.92-175.62) | 7.64(-0.02-20.33) | 5.75(1.07-12.73) | 0.69(0.10-1.69) | 53.33(-31.44-167.64) | 6.95(-0.31-18.66) |
| South Asia | 2.38(-0.65-6.27) | 0.21(0.03-0.48) | 49.40(-28.40-143.87) | 5.28(0.41-12.38) | 6.30(1.85-13.31) | 0.45(0.12-0.97) | 43.11(-30.89-134.80) | 4.83(0.25-11.61) |
| Southeast Asia | 10.26(2.47-19.01) | 0.68(0.16-1.38) | 238.60(46.69-450.35) | 16.48(3.28-33.09) | 16.52(4.03-31.83) | 2.03(0.36-4.38) | 222.08(38.71-425.92) | 14.45(2.00-29.81) |
| Southern Latin America | 4.91(0.74-10.01) | 1.73(0.37-3.59) | 94.98(-7.65-209.30) | 31.97(0.69-70.38) | 18.46(4.50-36.57) | 7.24(1.54-15.97) | 76.52(-13.64-180.01) | 24.73(-1.34-55.73) |
| Southern Sub-Saharan Africa | 12.35(4.68-21.10) | 4.49(2.13-7.68) | 268.55(81.26-478.19) | 91.25(41.17-160.36) | 16.48(4.09-33.48) | 6.88(1.22-16.36) | 252.08(71.84-448.66) | 84.37(38.38-146.43) |
| Tropical Latin America | 4.80(1.05-9.49) | 0.84(0.16-1.87) | 102.00(5.41-212.26) | 19.02(1.66-42.50) | 10.47(4.65-18.54) | 2.50(0.92-5.11) | 91.53(-0.85-196.03) | 16.52(0.34-37.42) |
| Western Europe | 4.39(1.53-8.42) | 1.76(0.37-3.56) | 91.81(25.51-172.98) | 29.60(0.78-62.38) | 27.73(11.23-49.66) | 8.96(2.05-19.05) | 64.08(10.38-129.61) | 20.63(-1.66-45.24) |
| Western Sub-Saharan Africa | 10.25(2.25-19.73) | 3.62(1.11-6.75) | 230.77(51.61-436.91) | 78.36(22.09-149.16) | 16.45(5.14-31.19) | 6.95(1.29-15.44) | 214.33(46.22-406.21) | 71.41(18.58-136.82) |

**eTable 4:** The number of Death, DALYs, YLDs, and YLLs of high alcohol use-related CVD in different age groups in 2021. DALYs, disability-adjusted life years; YLDs, years lived with disability; YLLs, years of life lost; CVD, cardiovascular diseases.

|  | **Death (95% UI)** | | **DALYs (95% UI)** | | **YLDs (95% UI)** | | **YLLs (95% UI)** | |
| --- | --- | --- | --- | --- | --- | --- | --- | --- |
| **Age Groups** | **Male** | **Female** | **Male** | **Female** | **Male** | **Female** | **Male** | **Female** |
| 15-19 years | -165.42(-320.68-16.87) | -18.46(-59.53-27.44) | -11677.72(-23343.12-2317.29) | -1326.46(-4514.07-2689.17) | 313.64(-514.5-1636.33) | 12.76(-598.84-868.6) | -11991.35(-23241.91-1214.81) | -1339.22(-4318.04-1990.87) |
| 20-24 years | -606.22(-1361.42-174.24) | -18.17(-111.68-85.08) | -39389.57(-92161.95-16172.73) | -659.55(-7775.68-9385.29) | 1596.73(26.21-6870.72) | 570.52(-708.19-3773.79) | -40986.31(-92034.07-11775.12) | -1230.07(-7556.27-5753.23) |
| 25-29 years | -662.93(-2088.85-702.92) | 75.07(-48.11-217.35) | -38317.16(-131531.3-54579.58) | 5885.51(-3248.43-18596.44) | 3288.42(-845.75-13335.17) | 1199.35(-929.86-5995.74) | -41605.58(-130920.06-43969.32) | 4686.15(-3032.39-13599) |
| 30-34 years | 683.47(-2062.54-3279.92) | 378.13(125.96-614.01) | 49207.25(-116889.3-209359.19) | 24516.12(6747.91-42142.52) | 10001.01(-131-25066.77) | 2754.14(-834.15-8816.25) | 39206.24(-119154.65-188964.82) | 21761.98(7218.18-35367.7) |
| 35-39 years | 2976.97(-1123.33-6723.38) | 815.07(385.16-1167.54) | 175382.91(-44566.68-387472.39) | 47485.94(20334.9-71446.41) | 18253.13(2669.95-38088.04) | 4473(-533.65-11480.14) | 157129.78(-59342.41-354927.41) | 43012.94(20326.35-61615.4) |
| 40-44 years | 6484.14(269.58-12518.34) | 1315.02(665.43-1936.98) | 339430.24(30474.53-633922.24) | 70106.2(33636.95-108371.33) | 29130.98(7054.71-54445.47) | 7164.24(671.12-16446.79) | 310299.26(12949.35-599036.33) | 62941.96(31861.92-92697.42) |
| 45-49 years | 11198.26(2345.77-20386.79) | 1869.35(894.7-2904.11) | 530009.6(126948.94-967487.41) | 91347.22(42296.47-143997.47) | 49015.04(13883.2-89158.42) | 10968.89(1948.2-23979.59) | 480994.56(100942.2-875368.86) | 80378.33(38505.33-124845.31) |
| 50-54 years | 17878.31(4247.2-32850.62) | 2495.28(1047.9-4236.1) | 754838.1(198650.58-1366788.11) | 110432.57(46579.06-184642.71) | 72002.02(22084.55-137145.84) | 15109.58(2640.22-32790.56) | 682836.08(162372.01-1254460.81) | 95322.98(40070.13-161774.44) |
| 55-59 years | 24771.55(6610.62-45271.98) | 3547.08(1633.8-5846.8) | 922591.34(267987.25-1673550.89) | 138118.42(63088.91-226813.79) | 92493.48(24510.85-179293.86) | 19417.8(3610.24-40747.33) | 830097.86(221132.76-1517444.85) | 118700.62(54645.28-195709.34) |
| 60-64 years | 32356.59(11229.96-56425.94) | 4420.42(1751.27-8058.63) | 1036071.97(354224.5-1782050.32) | 151345.21(62343.96-273758.54) | 102127.78(27647.12-198246.77) | 23781.42(4409-53396.84) | 933944.19(324370.17-1628786.29) | 127563.79(50542.02-232553.49) |
| 65-69 years | 41518.62(13183.91-73598.76) | 5181.79(1770.09-10208.13) | 1132246.97(378324.59-2001735.49) | 152978.77(56185.71-288268.22) | 122639.91(31775.48-238626.99) | 27075.35(6090.57-58971.23) | 1009607.06(320773.5-1789497.19) | 125903.42(43043.2-248068.34) |
| 70-74 years | 47809.47(14783.21-86533.33) | 6476.14(2004.98-12959.78) | 1079537.79(335808.78-1951585.04) | 158909.12(47203.52-311165.5) | 122853.06(33132.41-247223.53) | 29495.55(7298.85-63943.2) | 956684.73(295958.24-1731614.21) | 129413.57(40113.07-258932.74) |
| 75-79 years | 44169.57(13565.55-84316.29) | 7295.21(2247.04-14628.89) | 801899.39(250132.76-1522904.31) | 140666.96(42645.36-274718.11) | 96177.07(27574.77-194870.81) | 24658.53(6673.67-52896) | 705722.32(216665.09-1347358.16) | 116008.43(35731.07-232647.5) |
| 80-84 years | 37282.23(12315.19-71311.78) | 8817.3(2692.83-17940.36) | 38174.27(16901.26-68453.86) | 46870.73(16378.98-93891.81) | 64834.78(17830.83-132368.88) | 21034.12(5484.94-43403.36) | 465001.51(153639.6-889591.83) | 109526.19(33456.38-222828.91) |
| 85-89 years | 32809.66(12046.56-62522.79) | 10233.95(3339.29-20685.69) | 529836.29(172331.51-1020583.26) | 130560.31(41102.64-263669.81) | 33805.56(9940.81-68122.78) | 16345.56(4307.04-33646.21) | 325023.18(119394.72-619364.86) | 100920.27(32948.22-203996.55) |
| 90-94 years | 15557.53(6641.56-28811.62) | 9163.28(3433.39-18581.67) | 358828.73(132879.44-682886.87) | 117265.83(38597.79-234647.24) | 11702.31(3563.76-23313.59) | 8906.54(2295.22-18246.65) | 134266.57(57311.47-248648.42) | 78875.23(29556.37-159951.45) |
| 95+ years | 4328.88(1994.52-7841.05) | 5387.98(1879.49-10814.86) | 145968.89(63098.46-269606.55) | 87781.78(32518.99-176874.48) | 3062.03(841.17-6278.5) | 3456.8(777.81-7434.37) | 35112.24(16244.94-63550.04) | 43413.93(15220.02-87005.71) |

**eTable 5:** The rate of Death, DALYs, YLDs, and YLLs of high alcohol use-related CVD in different age groups in 2021. DALYs, disability-adjusted life years; YLDs, years lived with disability; YLLs, years of life lost; CVD, cardiovascular diseases.

|  | **Death Rate** **per 100,000, N (95% UI)** | | **DALYs Rate** **per 100,000, N (95% UI)** | | **YLDS Rate** **per 100,000, N (95% UI)** | | **YLLS Rate** **per 100,000, N (95% UI)** | |
| --- | --- | --- | --- | --- | --- | --- | --- | --- |
| **Age Groups** | **Male** | **Female** | **Male** | **Female** | **Male** | **Female** | **Male** | **Female** |
| 15-19 years | -0.05(-0.1-0.01) | -0.01(-0.02-0.01) | -3.65(-7.29-0.72) | -0.44(-1.49-0.89) | 0.1(-0.16-0.51) | 0(-0.2-0.29) | -3.74(-7.26-0.38) | -0.44(-1.42-0.66) |
| 20-24 years | -0.2(-0.45-0.06) | -0.01(-0.04-0.03) | -12.98(-30.38-5.33) | -0.22(-2.65-3.19) | 0.53(0.01-2.26) | 0.19(-0.24-1.28) | -13.51(-30.33-3.88) | -0.42(-2.57-1.96) |
| 25-29 years | -0.22(-0.7-0.24) | 0.03(-0.02-0.07) | -12.89(-44.23-18.35) | 2.02(-1.12-6.39) | 1.11(-0.28-4.48) | 0.41(-0.32-2.06) | -13.99(-44.03-14.79) | 1.61(-1.04-4.67) |
| 30-34 years | 0.22(-0.68-1.07) | 0.13(0.04-0.21) | 16.1(-38.26-68.52) | 8.2(2.26-14.1) | 3.27(-0.04-8.2) | 0.92(-0.28-2.95) | 12.83(-39-61.84) | 7.28(2.41-11.83) |
| 35-39 years | 1.05(-0.4-2.38) | 0.29(0.14-0.42) | 61.96(-15.74-136.89) | 17.09(7.32-25.72) | 6.45(0.94-13.46) | 1.61(-0.19-4.13) | 55.51(-20.96-125.39) | 15.48(7.32-22.18) |
| 40-44 years | 2.57(0.11-4.96) | 0.53(0.27-0.78) | 134.61(12.09-251.4) | 28.26(13.56-43.68) | 11.55(2.8-21.59) | 2.89(0.27-6.63) | 123.06(5.14-237.56) | 25.37(12.84-37.36) |
| 45-49 years | 4.71(0.99-8.57) | 0.79(0.38-1.23) | 222.82(53.37-406.74) | 38.77(17.95-61.11) | 20.61(5.84-37.48) | 4.65(0.83-10.18) | 202.22(42.44-368.02) | 34.11(16.34-52.98) |
| 50-54 years | 8.05(1.91-14.8) | 1.12(0.47-1.9) | 340.05(89.49-615.72) | 49.53(20.89-82.82) | 32.44(9.95-61.78) | 6.78(1.18-14.71) | 307.61(73.15-565.12) | 42.76(17.97-72.56) |
| 55-59 years | 12.72(3.39-23.25) | 1.76(0.81-2.91) | 473.79(137.62-859.45) | 68.71(31.39-112.84) | 47.5(12.59-92.08) | 9.66(1.8-20.27) | 426.29(113.56-779.28) | 59.05(27.19-97.37) |
| 60-64 years | 20.8(7.22-36.28) | 2.69(1.06-4.9) | 666.13(227.74-1145.74) | 92(37.9-166.41) | 65.66(17.78-127.46) | 14.46(2.68-32.46) | 600.46(208.55-1047.2) | 77.54(30.72-141.36) |
| 65-69 years | 31.49(10-55.83) | 3.6(1.23-7.09) | 858.85(286.97-1518.38) | 106.23(39.02-200.17) | 93.03(24.1-181.01) | 18.8(4.23-40.95) | 765.82(243.32-1357.39) | 87.43(29.89-172.26) |
| 70-74 years | 49.6(15.34-89.77) | 5.92(1.83-11.84) | 1119.95(348.38-2024.65) | 145.19(43.13-284.3) | 127.45(34.37-256.48) | 26.95(6.67-58.42) | 992.5(307.04-1796.44) | 118.24(36.65-236.58) |
| 75-79 years | 73.88(22.69-141.03) | 10.12(3.12-20.29) | 1341.27(418.38-2547.23) | 195.11(59.15-381.04) | 160.87(46.12-325.94) | 34.2(9.26-73.37) | 1180.4(362.4-2253.61) | 160.9(49.56-322.68) |
| 80-84 years | 101.72(33.6-194.57) | 17.31(5.29-35.22) | 2524.68(1117.78-4527.25) | 1190.14(415.89-2384.09) | 176.89(48.65-361.15) | 41.3(10.77-85.22) | 1268.7(419.19-2427.14) | 215.05(65.69-437.51) |
| 85-89 years | 190.17(69.82-362.4) | 35.95(11.73-72.66) | 1445.59(470.18-2784.53) | 256.35(80.7-517.7) | 195.94(57.62-394.85) | 57.42(15.13-118.18) | 1883.9(692.04-3589.97) | 354.49(115.73-716.55) |
| 90-94 years | 266.92(113.95-494.32) | 75.98(28.47-154.07) | 2079.85(770.2-3958.15) | 411.91(135.58-824.22) | 200.78(61.14-399.99) | 73.85(19.03-151.29) | 2303.62(983.3-4266.08) | 653.98(245.06-1326.2) |
| 95+ years | 286.29(131.91-518.57) | 136.81(47.72-274.61) | 2504.4(1082.59-4625.66) | 727.82(269.62-1466.51) | 202.51(55.63-415.23) | 87.77(19.75-188.77) | 2322.17(1074.37-4202.93) | 1102.36(386.46-2209.24) |

**eTable 6:** ASMR for five types of high alcohol use-related CVD in both sexes combined globally, 1990-2021. ASMR, age-standardized mortality rate; CVD, cardiovascular diseases.

| **Year** | **Cardiovascular diseases** | **Stroke** | **Atrial fibrillation and flutter** | **Cardiomyopathy and myocarditis** | **Hypertensive heart disease** | **Ischemic heart disease** |
| --- | --- | --- | --- | --- | --- | --- |
| 1990 | 7.23(2.43-14.03) | 7.2(1.4-14.66) | 0.15(0.1-0.2) | 1.18(1.11-1.25) | 1.12(0.72-1.61) | -2.42(-4.2--0.23) |
| 1991 | 7.14(2.38-13.52) | 7.08(1.41-14.39) | 0.15(0.1-0.2) | 1.2(1.13-1.26) | 1.11(0.7-1.58) | -2.4(-4.15--0.27) |
| 1992 | 7.12(2.44-13.68) | 7.02(1.44-14.13) | 0.15(0.1-0.2) | 1.26(1.19-1.32) | 1.1(0.69-1.55) | -2.41(-4.14--0.3) |
| 1993 | 7.23(2.5-13.95) | 7.05(1.43-14.27) | 0.15(0.1-0.2) | 1.39(1.34-1.45) | 1.08(0.68-1.52) | -2.45(-4.24--0.3) |
| 1994 | 7.29(2.65-13.88) | 7.02(1.39-14.14) | 0.15(0.1-0.2) | 1.5(1.45-1.57) | 1.06(0.67-1.5) | -2.44(-4.24--0.28) |
| 1995 | 7.23(2.58-13.74) | 6.95(1.41-13.94) | 0.15(0.11-0.2) | 1.49(1.44-1.56) | 1.03(0.65-1.46) | -2.41(-4.24--0.28) |
| 1996 | 7.02(2.51-13.26) | 6.77(1.39-13.56) | 0.15(0.11-0.2) | 1.43(1.38-1.49) | 1.01(0.65-1.43) | -2.34(-4.07--0.27) |
| 1997 | 6.75(2.35-12.74) | 6.56(1.33-12.89) | 0.15(0.1-0.2) | 1.34(1.28-1.4) | 0.99(0.61-1.41) | -2.29(-3.96--0.28) |
| 1998 | 6.55(2.24-12.52) | 6.4(1.33-12.62) | 0.15(0.1-0.19) | 1.29(1.22-1.35) | 0.97(0.6-1.39) | -2.25(-3.89--0.32) |
| 1999 | 6.49(2.22-12.27) | 6.29(1.29-12.52) | 0.15(0.11-0.2) | 1.33(1.28-1.39) | 0.96(0.59-1.39) | -2.25(-3.88--0.32) |
| 2000 | 6.55(2.32-12.19) | 6.21(1.32-12.18) | 0.15(0.11-0.2) | 1.43(1.37-1.49) | 0.98(0.6-1.41) | -2.21(-3.79--0.31) |
| 2001 | 6.56(2.35-12.23) | 6.15(1.27-12.11) | 0.15(0.11-0.2) | 1.46(1.4-1.52) | 0.97(0.6-1.41) | -2.18(-3.75--0.32) |
| 2002 | 6.67(2.5-12.21) | 6.12(1.29-11.94) | 0.15(0.11-0.2) | 1.59(1.54-1.65) | 0.97(0.61-1.4) | -2.16(-3.72--0.3) |
| 2003 | 6.8(2.62-12.3) | 6.09(1.29-12.05) | 0.15(0.11-0.2) | 1.72(1.67-1.78) | 0.97(0.61-1.4) | -2.14(-3.66--0.39) |
| 2004 | 6.82(2.8-12.04) | 5.94(1.26-11.67) | 0.15(0.11-0.2) | 1.85(1.8-1.91) | 0.95(0.6-1.36) | -2.08(-3.57--0.4) |
| 2005 | 7.11(3.17-12.26) | 5.8(1.25-11.29) | 0.15(0.11-0.2) | 2.25(2.21-2.32) | 0.95(0.6-1.36) | -2.04(-3.52--0.38) |
| 2006 | 6.77(3.04-11.58) | 5.48(1.19-10.7) | 0.15(0.11-0.2) | 2.15(2.09-2.2) | 0.94(0.59-1.34) | -1.96(-3.35--0.42) |
| 2007 | 6.55(2.9-11.29) | 5.35(1.15-10.45) | 0.15(0.11-0.2) | 2.02(1.96-2.07) | 0.94(0.59-1.35) | -1.92(-3.25--0.43) |
| 2008 | 6.53(2.89-11.23) | 5.33(1.2-10.33) | 0.16(0.11-0.2) | 1.98(1.92-2.03) | 0.96(0.61-1.38) | -1.89(-3.21--0.43) |
| 2009 | 6.33(2.68-10.89) | 5.26(1.2-10.17) | 0.16(0.11-0.2) | 1.8(1.73-1.85) | 0.98(0.62-1.41) | -1.86(-3.09--0.41) |
| 2010 | 6.16(2.57-10.68) | 5.2(1.16-10.23) | 0.16(0.11-0.2) | 1.66(1.6-1.71) | 0.99(0.62-1.42) | -1.84(-3.04--0.41) |
| 2011 | 5.69(2.19-9.99) | 5.05(1.14-9.97) | 0.16(0.11-0.2) | 1.29(1.23-1.35) | 0.99(0.62-1.42) | -1.8(-2.96--0.39) |
| 2012 | 5.45(2.02-9.76) | 4.95(1.12-9.64) | 0.16(0.11-0.2) | 1.14(1.08-1.19) | 0.99(0.63-1.43) | -1.78(-2.91--0.38) |
| 2013 | 5.28(1.93-9.58) | 4.82(1.08-9.44) | 0.16(0.12-0.2) | 1.05(1-1.1) | 1(0.63-1.43) | -1.75(-2.85--0.42) |
| 2014 | 5.16(1.87-9.35) | 4.72(1.04-9.14) | 0.16(0.12-0.2) | 1(0.95-1.05) | 1(0.63-1.44) | -1.72(-2.8--0.41) |
| 2015 | 5.06(1.85-9.21) | 4.64(1.05-9.23) | 0.16(0.12-0.2) | 0.94(0.9-0.99) | 1(0.64-1.45) | -1.69(-2.74--0.39) |
| 2016 | 4.99(1.83-8.98) | 4.59(1.03-9.18) | 0.16(0.12-0.2) | 0.9(0.85-0.94) | 1.01(0.63-1.47) | -1.66(-2.67--0.39) |
| 2017 | 4.85(1.74-8.73) | 4.48(1-8.61) | 0.16(0.12-0.2) | 0.83(0.77-0.88) | 1(0.63-1.46) | -1.62(-2.56--0.38) |
| 2018 | 4.77(1.69-8.66) | 4.42(1.02-8.54) | 0.16(0.12-0.2) | 0.79(0.74-0.85) | 0.99(0.62-1.41) | -1.59(-2.57--0.38) |
| 2019 | 4.71(1.68-8.6) | 4.37(0.98-8.63) | 0.16(0.12-0.2) | 0.77(0.71-0.83) | 0.98(0.62-1.41) | -1.56(-2.51--0.35) |
| 2020 | 4.69(1.72-8.6) | 4.33(0.98-8.53) | 0.15(0.11-0.19) | 0.76(0.68-0.82) | 0.98(0.61-1.38) | -1.54(-2.46--0.35) |
| 2021 | 4.6(1.62-8.42) | 4.3(1-8.39) | 0.15(0.11-0.19) | 0.74(0.65-0.8) | 0.94(0.57-1.34) | -1.53(-2.45--0.35) |

**eTable 7:** ASDR for five types of high alcohol use-related CVD in both sexes combined globally, 1990-2021. DALYs, disability-adjusted life years; ASDR, age-standardized rate of DALYs; CVD, cardiovascular diseases.

| **Year** | **Cardiovascular diseases** | **Stroke** | **Atrial fibrillation and flutter** | **Cardiomyopathy and myocarditis** | **Hypertensive heart disease** | **Ischemic heart disease** |
| --- | --- | --- | --- | --- | --- | --- |
| 1990 | 156.46(44.83-295.59) | 154.83(33.98-299.48) | 4.4(2.95-5.79) | 35.87(33.46-37.95) | 23.75(15.16-34.43) | -62.38(-97.67--14.96) |
| 1991 | 155.49(44.92-290.66) | 153.03(33.52-293.56) | 4.37(2.93-5.73) | 36.69(34.43-38.74) | 23.54(15.03-33.07) | -62.14(-96.98--15.42) |
| 1992 | 156.92(46.86-292.91) | 152.57(35.14-292.68) | 4.35(2.92-5.69) | 39.28(37.29-41.16) | 23.24(14.96-32.89) | -62.52(-98.51--15.58) |
| 1993 | 162.04(50.02-302.4) | 154.08(34.55-301.46) | 4.34(2.91-5.68) | 44.82(43.38-46.69) | 22.91(14.73-32) | -64.1(-101.04--15.59) |
| 1994 | 166.3(54.95-307.32) | 154.15(35.13-298.31) | 4.33(2.91-5.64) | 49.68(48.15-51.93) | 22.47(14.57-31.46) | -64.33(-102.82--15.89) |
| 1995 | 165.29(54.99-300.84) | 152.65(34.42-292.04) | 4.33(2.91-5.67) | 49.5(47.98-51.78) | 21.94(13.96-30.94) | -63.13(-101.3--15.84) |
| 1996 | 160.31(53.22-294.45) | 148.65(33.48-284.65) | 4.32(2.92-5.65) | 47.01(45.66-48.98) | 21.42(13.8-29.96) | -61.08(-97.55--16.47) |
| 1997 | 153.6(49.02-283.34) | 144.04(32.76-273.58) | 4.3(2.94-5.65) | 43.68(41.82-45.63) | 20.79(13.17-29.47) | -59.22(-94.36--17.17) |
| 1998 | 149.03(46.62-274.02) | 140.37(31.95-270.21) | 4.3(2.95-5.65) | 42.18(40.02-44.12) | 20.29(12.87-28.73) | -58.11(-93.27--16.71) |
| 1999 | 148.64(46.92-273.05) | 138.28(31.22-264.87) | 4.31(2.99-5.63) | 44.05(42.23-46.05) | 20.11(12.82-28.48) | -58.11(-93.08--16.97) |
| 2000 | 152.48(52.19-273.92) | 136.99(31.38-260.62) | 4.3(3.01-5.63) | 48.49(46.77-50.4) | 20.22(12.71-28.8) | -57.53(-92.22--16.79) |
| 2001 | 154.31(53.94-273.03) | 135.79(31.08-256.26) | 4.3(3.02-5.61) | 50.65(48.85-52.57) | 20.13(12.53-28.69) | -56.57(-90.94--16.32) |
| 2002 | 159.62(59.29-276.52) | 135.24(32.52-256) | 4.33(3.05-5.65) | 55.99(54.39-58.06) | 20.05(12.63-28.45) | -55.99(-89.36--15.79) |
| 2003 | 164.92(64.46-281.54) | 134.67(30.6-260.37) | 4.37(3.1-5.67) | 61.34(59.73-63.41) | 19.91(12.72-28.01) | -55.36(-88.74--15.41) |
| 2004 | 168.9(71.91-284.63) | 131.98(30.89-251.43) | 4.38(3.11-5.68) | 66.98(65.26-69) | 19.55(12.38-27.49) | -53.98(-86.37--14.95) |
| 2005 | 183.03(88.18-295.73) | 129.37(31.53-245.05) | 4.39(3.13-5.68) | 83.01(81.59-85.55) | 19.44(12.38-27.35) | -53.19(-85.04--15.25) |
| 2006 | 174.83(84.56-285.04) | 123.23(29.91-232.58) | 4.38(3.14-5.66) | 79.05(77.16-80.82) | 19.22(12.4-27.05) | -51.05(-81.36--13.99) |
| 2007 | 167.98(79.63-272.7) | 120.52(28.78-228.65) | 4.39(3.15-5.68) | 73.67(71.67-75.52) | 19.29(12.52-27.04) | -49.9(-79--13.4) |
| 2008 | 166.38(78.09-267.31) | 120.19(29.27-224.28) | 4.41(3.17-5.7) | 71.55(69.83-73.52) | 19.57(12.75-27.5) | -49.34(-77.5--13.19) |
| 2009 | 158.82(70.6-258.62) | 118.58(28.63-223.42) | 4.42(3.19-5.73) | 64.14(62.03-65.89) | 19.8(12.95-28.07) | -48.12(-75.29--12.64) |
| 2010 | 152.86(65.82-256.23) | 117.31(28.66-223.12) | 4.43(3.2-5.76) | 58.65(56.75-60.58) | 19.99(13.04-28.21) | -47.51(-74.01--12.61) |
| 2011 | 137.32(52.32-237.35) | 114.43(28.01-217.67) | 4.42(3.19-5.76) | 45.1(42.99-46.78) | 19.91(12.91-28.01) | -46.54(-71.72--12.24) |
| 2012 | 130.25(46.23-229.63) | 112.3(27.46-213.24) | 4.41(3.2-5.72) | 39.33(37.52-40.87) | 19.93(13.07-28.19) | -45.73(-70.9--12.08) |
| 2013 | 125.14(42.36-219.57) | 109.56(26.52-208.66) | 4.39(3.19-5.7) | 36.11(34.45-37.74) | 19.96(12.93-28.23) | -44.87(-69.89--11.7) |
| 2014 | 122.09(42.01-216.57) | 107.19(25.67-199.81) | 4.38(3.19-5.67) | 34.46(33.05-36.04) | 19.96(12.87-28.18) | -43.89(-67.51--11.46) |
| 2015 | 119.35(40.48-211.98) | 105.53(25.4-202.71) | 4.38(3.2-5.66) | 32.34(30.86-33.92) | 20.05(12.88-28.48) | -42.96(-66.86--11.08) |
| 2016 | 117.32(38.32-209.14) | 104.39(25.59-199.43) | 4.38(3.19-5.64) | 30.69(29.14-32.25) | 20.12(13-28.53) | -42.25(-65.6--10.86) |
| 2017 | 113.62(36.3-201.32) | 102.07(24.73-193.17) | 4.38(3.2-5.63) | 28.36(26.39-29.9) | 20(12.82-28.57) | -41.18(-63.28--10.98) |
| 2018 | 111.51(35.53-198.8) | 100.74(24.95-190.16) | 4.37(3.21-5.62) | 27.14(25.36-28.71) | 19.84(12.77-27.75) | -40.58(-62.92--10.59) |
| 2019 | 110.33(34.89-202.65) | 99.66(24.44-194.39) | 4.35(3.18-5.65) | 26.47(24.39-28.3) | 19.69(12.76-27.71) | -39.83(-61.56--10.36) |
| 2020 | 109.62(36.05-197.58) | 98.7(23.79-190.4) | 4.33(3.12-5.59) | 25.97(23.2-27.89) | 19.57(12.49-27.18) | -38.96(-60.61--10.09) |
| 2021 | 107.59(33.46-191.01) | 97.89(23.83-187.71) | 4.32(3.15-5.57) | 25.34(22.38-27.45) | 18.86(12.17-26.66) | -38.81(-60.37--9.97) |

**eTable 8:** Age-Standardized Rate of YLDs for five types of high alcohol use-related CVD in both sexes combined globally, 1990-2021. YLDs, years lived with disability; CVD, cardiovascular diseases.

| **year** | **Cardiovascular diseases** | **Stroke** | **Atrial fibrillation and flutter** | **Cardiomyopathy and myocarditis** | **Hypertensive heart disease** | **Ischemic heart disease** |
| --- | --- | --- | --- | --- | --- | --- |
| 1990 | 12.82(3.34-25.63) | 10.2(0.89-23.02) | 2.3(1.29-3.43) | 0.69(0.47-0.96) | 0.65(0.37-1.04) | -1.03(-1.79--0.26) |
| 1991 | 12.8(3.32-25.54) | 10.2(0.9-23.02) | 2.28(1.28-3.39) | 0.69(0.47-0.95) | 0.66(0.37-1.05) | -1.03(-1.78--0.26) |
| 1992 | 12.78(3.3-25.51) | 10.2(0.9-23.05) | 2.26(1.27-3.35) | 0.69(0.46-0.95) | 0.67(0.38-1.06) | -1.03(-1.79--0.26) |
| 1993 | 12.75(3.29-25.47) | 10.18(0.89-22.99) | 2.24(1.26-3.31) | 0.69(0.46-0.94) | 0.67(0.38-1.07) | -1.03(-1.79--0.26) |
| 1994 | 12.71(3.29-25.44) | 10.16(0.88-22.97) | 2.23(1.26-3.29) | 0.69(0.46-0.94) | 0.67(0.39-1.06) | -1.04(-1.79--0.26) |
| 1995 | 12.68(3.3-25.44) | 10.13(0.87-23) | 2.22(1.26-3.28) | 0.68(0.47-0.95) | 0.67(0.39-1.06) | -1.04(-1.78--0.27) |
| 1996 | 12.63(3.3-25.34) | 10.08(0.87-22.89) | 2.22(1.28-3.25) | 0.69(0.47-0.95) | 0.67(0.39-1.07) | -1.04(-1.79--0.26) |
| 1997 | 12.55(3.31-25.24) | 10.01(0.87-22.79) | 2.22(1.28-3.22) | 0.69(0.47-0.95) | 0.67(0.38-1.07) | -1.04(-1.79--0.25) |
| 1998 | 12.47(3.31-25.06) | 9.91(0.86-22.6) | 2.22(1.3-3.22) | 0.7(0.48-0.97) | 0.67(0.39-1.07) | -1.04(-1.78--0.25) |
| 1999 | 12.39(3.33-24.95) | 9.82(0.86-22.47) | 2.23(1.32-3.22) | 0.71(0.48-0.97) | 0.67(0.39-1.07) | -1.04(-1.78--0.25) |
| 2000 | 12.33(3.34-24.85) | 9.76(0.85-22.36) | 2.22(1.32-3.21) | 0.71(0.49-0.98) | 0.67(0.39-1.07) | -1.03(-1.78--0.25) |
| 2001 | 12.3(3.36-24.76) | 9.71(0.85-22.25) | 2.23(1.32-3.22) | 0.72(0.49-0.98) | 0.68(0.39-1.08) | -1.03(-1.77--0.25) |
| 2002 | 12.29(3.38-24.67) | 9.67(0.84-22.17) | 2.24(1.33-3.22) | 0.72(0.5-1) | 0.68(0.4-1.09) | -1.03(-1.76--0.24) |
| 2003 | 12.29(3.43-24.58) | 9.63(0.84-22.18) | 2.25(1.35-3.24) | 0.73(0.5-1) | 0.69(0.4-1.11) | -1.02(-1.75--0.24) |
| 2004 | 12.28(3.47-24.61) | 9.6(0.84-22.17) | 2.26(1.36-3.25) | 0.73(0.5-1) | 0.7(0.41-1.12) | -1.01(-1.73--0.24) |
| 2005 | 12.26(3.48-24.59) | 9.56(0.83-22.13) | 2.26(1.37-3.26) | 0.73(0.5-1) | 0.71(0.42-1.14) | -1.01(-1.72--0.24) |
| 2006 | 12.26(3.5-24.57) | 9.55(0.84-22.1) | 2.26(1.37-3.27) | 0.73(0.5-1) | 0.72(0.43-1.15) | -1(-1.7--0.24) |
| 2007 | 12.3(3.53-24.69) | 9.58(0.85-22.17) | 2.26(1.36-3.27) | 0.72(0.5-0.99) | 0.74(0.44-1.17) | -1(-1.7--0.23) |
| 2008 | 12.37(3.58-24.79) | 9.62(0.86-22.26) | 2.27(1.37-3.27) | 0.72(0.49-0.99) | 0.76(0.45-1.2) | -1(-1.69--0.23) |
| 2009 | 12.43(3.61-24.93) | 9.67(0.87-22.38) | 2.27(1.37-3.27) | 0.71(0.49-0.98) | 0.77(0.46-1.22) | -0.99(-1.67--0.23) |
| 2010 | 12.47(3.64-25.01) | 9.69(0.87-22.44) | 2.27(1.37-3.27) | 0.7(0.48-0.97) | 0.78(0.47-1.24) | -0.99(-1.68--0.23) |
| 2011 | 12.46(3.66-24.98) | 9.68(0.88-22.39) | 2.27(1.36-3.26) | 0.69(0.47-0.95) | 0.79(0.48-1.25) | -0.98(-1.67--0.22) |
| 2012 | 12.44(3.64-24.91) | 9.67(0.88-22.33) | 2.26(1.36-3.25) | 0.68(0.47-0.93) | 0.81(0.48-1.27) | -0.97(-1.65--0.22) |
| 2013 | 12.41(3.61-24.82) | 9.65(0.88-22.24) | 2.25(1.35-3.23) | 0.66(0.45-0.91) | 0.82(0.49-1.29) | -0.97(-1.64--0.22) |
| 2014 | 12.38(3.59-24.82) | 9.62(0.88-22.2) | 2.24(1.34-3.22) | 0.65(0.45-0.89) | 0.83(0.49-1.3) | -0.96(-1.64--0.22) |
| 2015 | 12.35(3.57-24.7) | 9.6(0.87-22.07) | 2.24(1.34-3.21) | 0.64(0.44-0.87) | 0.84(0.5-1.32) | -0.96(-1.63--0.21) |
| 2016 | 12.31(3.53-24.63) | 9.56(0.87-21.96) | 2.23(1.33-3.21) | 0.62(0.43-0.85) | 0.84(0.5-1.32) | -0.95(-1.62--0.21) |
| 2017 | 12.26(3.53-24.5) | 9.52(0.86-21.87) | 2.24(1.34-3.21) | 0.6(0.41-0.83) | 0.85(0.5-1.34) | -0.94(-1.61--0.21) |
| 2018 | 12.22(3.5-24.35) | 9.49(0.85-21.71) | 2.24(1.33-3.23) | 0.59(0.4-0.8) | 0.85(0.5-1.34) | -0.94(-1.6--0.21) |
| 2019 | 12.2(3.5-24.22) | 9.47(0.85-21.55) | 2.24(1.33-3.22) | 0.58(0.39-0.79) | 0.85(0.51-1.35) | -0.93(-1.59--0.21) |
| 2020 | 12.17(3.48-24.21) | 9.45(0.84-21.58) | 2.25(1.33-3.24) | 0.56(0.38-0.77) | 0.84(0.5-1.34) | -0.92(-1.57--0.21) |
| 2021 | 12.18(3.48-24.06) | 9.45(0.85-21.18) | 2.27(1.35-3.25) | 0.54(0.36-0.75) | 0.85(0.5-1.36) | -0.92(-1.57--0.21) |

**eTable 9:** Age-Standardized Rate of YLLs for five types of high alcohol use-related CVD in both sexes combined globally, 1990-2021. YLLs, years of life lost; CVD, cardiovascular diseases.

| **year** | **Cardiovascular diseases** | **Stroke** | **Atrial fibrillation and flutter** | **Cardiomyopathy and myocarditis** | **Hypertensive heart disease** | **Ischemic heart disease** |
| --- | --- | --- | --- | --- | --- | --- |
| 1990 | 143.64(39.6-270.64) | 144.63(31.98-275.44) | 2.1(1.49-2.71) | 35.17(32.66-37.28) | 23.1(14.65-33.43) | -61.35(-96.11--14.55) |
| 1991 | 142.69(39.33-264.27) | 142.83(32.14-272.34) | 2.09(1.5-2.69) | 36(33.73-38.02) | 22.88(14.61-32.1) | -61.12(-95.36--15.16) |
| 1992 | 144.13(41.79-270.81) | 142.37(34.01-271.79) | 2.09(1.51-2.7) | 38.6(36.54-40.51) | 22.57(14.42-31.83) | -61.49(-97.15--15.33) |
| 1993 | 149.29(44.62-283.84) | 143.9(33.69-280.97) | 2.1(1.53-2.71) | 44.13(42.68-45.84) | 22.24(14.23-31.04) | -63.07(-99.38--15.33) |
| 1994 | 153.59(49.27-284.06) | 143.99(34.14-278.11) | 2.1(1.53-2.7) | 48.99(47.47-51.07) | 21.8(13.99-30.5) | -63.29(-101.05--15.59) |
| 1995 | 152.61(48.44-278.21) | 142.52(33.11-273.23) | 2.1(1.54-2.7) | 48.82(47.41-50.93) | 21.27(13.53-29.89) | -62.09(-99.48--15.58) |
| 1996 | 147.69(47.05-271.82) | 138.57(32.51-264.13) | 2.1(1.54-2.7) | 46.32(44.96-48.18) | 20.74(13.37-29.02) | -60.04(-95.98--16.08) |
| 1997 | 141.04(43.15-263.74) | 134.03(32.29-256.58) | 2.08(1.53-2.67) | 42.99(41.12-44.86) | 20.12(12.55-28.47) | -58.18(-92.67--16.87) |
| 1998 | 136.57(41.23-252.11) | 130.46(31.85-251.47) | 2.08(1.52-2.65) | 41.48(39.3-43.32) | 19.62(12.37-27.78) | -57.07(-91.58--16.41) |
| 1999 | 136.25(42.12-253.01) | 128.46(30.99-247.9) | 2.08(1.53-2.65) | 43.34(41.48-45.29) | 19.44(12.22-27.71) | -57.07(-91.47--16.66) |
| 2000 | 140.15(47.09-255.61) | 127.24(30.35-243.09) | 2.08(1.52-2.65) | 47.78(46.04-49.75) | 19.55(12.25-27.82) | -56.49(-90.36--16.48) |
| 2001 | 142(49.83-257.93) | 126.08(30.26-239.69) | 2.08(1.53-2.65) | 49.93(48.16-51.81) | 19.45(12.03-27.68) | -55.54(-89.15--16.02) |
| 2002 | 147.33(55.07-255.61) | 125.57(31.18-239.74) | 2.09(1.54-2.66) | 55.27(53.64-57.13) | 19.36(12.17-27.58) | -54.97(-87.73--15.5) |
| 2003 | 152.63(60.16-263.17) | 125.04(30.68-241.52) | 2.12(1.55-2.7) | 60.61(59.01-62.41) | 19.21(12.18-26.92) | -54.34(-87.02--15.12) |
| 2004 | 156.62(66.53-264.92) | 122.38(29.47-235.31) | 2.12(1.58-2.69) | 66.25(64.53-68.09) | 18.84(11.84-26.51) | -52.97(-84.55--14.67) |
| 2005 | 170.76(81.97-277.44) | 119.8(30.06-228.58) | 2.13(1.59-2.7) | 82.27(80.9-84.65) | 18.73(11.81-26.35) | -52.18(-83.23--14.96) |
| 2006 | 162.57(78.62-266.1) | 113.68(28.33-215.7) | 2.12(1.6-2.69) | 78.32(76.39-80.05) | 18.5(11.76-26.12) | -50.05(-79.58--13.72) |
| 2007 | 155.68(73.88-256.12) | 110.94(27.49-208.52) | 2.13(1.61-2.7) | 72.95(70.98-74.57) | 18.55(11.87-26.12) | -48.9(-77.26--13.13) |
| 2008 | 154.01(71.72-248.37) | 110.57(27.89-206.9) | 2.14(1.62-2.71) | 70.83(69.14-72.63) | 18.81(12.15-26.39) | -48.35(-75.91--12.92) |
| 2009 | 146.39(64.88-242.99) | 108.92(27.15-207.03) | 2.15(1.63-2.7) | 63.43(61.29-65.07) | 19.03(12.37-26.95) | -47.13(-73.9--12.37) |
| 2010 | 140.4(59.46-235.64) | 107.61(27.17-203.18) | 2.16(1.63-2.71) | 57.95(56.05-59.78) | 19.2(12.38-27.01) | -46.53(-72.51--12.34) |
| 2011 | 124.86(46.43-221.22) | 104.75(26.67-199.49) | 2.15(1.64-2.69) | 44.41(42.3-46.08) | 19.11(12.23-27.02) | -45.56(-70.36--11.97) |
| 2012 | 117.81(40.86-208.88) | 102.63(25.27-193.3) | 2.15(1.64-2.7) | 38.65(36.83-40.22) | 19.13(12.36-27.01) | -44.75(-69.24--11.82) |
| 2013 | 112.73(38.52-205.82) | 99.91(26.18-189.7) | 2.14(1.63-2.68) | 35.45(33.75-37.04) | 19.14(12.21-27.16) | -43.91(-68.48--11.43) |
| 2014 | 109.71(37.54-199.16) | 97.57(24.31-182.15) | 2.13(1.63-2.67) | 33.81(32.4-35.38) | 19.13(12.33-27.1) | -42.93(-66.17--11.2) |
| 2015 | 107(36.04-193.28) | 95.94(23.67-181.63) | 2.15(1.64-2.69) | 31.7(30.18-33.31) | 19.22(12.37-27.1) | -42.01(-65.42--10.81) |
| 2016 | 105.02(34.62-189.62) | 94.83(23.01-179.03) | 2.15(1.63-2.69) | 30.07(28.49-31.59) | 19.27(12.5-27.29) | -41.3(-64.04--10.6) |
| 2017 | 101.36(33.28-184.53) | 92.55(23.1-174.71) | 2.14(1.62-2.67) | 27.75(25.79-29.39) | 19.16(12.31-27.21) | -40.24(-61.66--10.75) |
| 2018 | 99.28(32-178.06) | 91.25(23.24-170.53) | 2.13(1.62-2.65) | 26.55(24.84-28.16) | 18.99(12.3-26.71) | -39.64(-61.42--10.35) |
| 2019 | 98.13(30.84-179.69) | 90.19(23.01-174.27) | 2.1(1.6-2.66) | 25.89(23.81-27.66) | 18.83(12.28-26.53) | -38.89(-60.01--10.13) |
| 2020 | 97.44(32.59-179.44) | 89.26(21.68-170.97) | 2.08(1.58-2.59) | 25.41(22.64-27.28) | 18.72(11.95-25.99) | -38.03(-58.89--9.87) |
| 2021 | 95.41(30.03-172.02) | 88.44(22.34-167.25) | 2.06(1.56-2.56) | 24.8(21.82-26.88) | 18.01(11.58-25.3) | -37.89(-58.9--9.71) |
